# Impaired proactive cognitive control in Parkinson’s disease

**DOI:** 10.1093/braincomms/fcad327

**Published:** 2023-11-30

**Authors:** Julius Kricheldorff, Julia Ficke, Stefan Debener, Karsten Witt

**Affiliations:** Department of Neurology, School of Medicine and Health Science, Carl von Ossietzky University of Oldenburg, 26046 Oldenburg, Germany; Department of Neurology, School of Medicine and Health Science, Carl von Ossietzky University of Oldenburg, 26046 Oldenburg, Germany; Research Center of Neurosensory Science, Carl von Ossietzky University of Oldenburg, 26129 Oldenburg, Germany; Neuropsychology Lab, Department of Psychology, Carl von Ossietzky University of Oldenburg, 26129 Oldenburg, Germany; Cluster of Excellence Hearing4all, Carl von Ossietzky University of Oldenburg, 26129 Oldenburg, Germany; Department of Neurology, School of Medicine and Health Science, Carl von Ossietzky University of Oldenburg, 26046 Oldenburg, Germany; Research Center of Neurosensory Science, Carl von Ossietzky University of Oldenburg, 26129 Oldenburg, Germany; Department of Neurology, Evangelical Hospital, 26121 Oldenburg, Germany

**Keywords:** Parkinson’s disease, adaptive control, proactive control, reactive control, theta

## Abstract

Adaptive control has been studied in Parkinson’s disease mainly in the context of proactive control and with mixed results. We compared reactive- and proactive control in 30 participants with Parkinson’s disease to 30 age matched healthy control participants. The electroencephalographic activity of the participants was recorded over 128 channels while they performed a numerical Stroop task, in which we controlled for confounding stimulus-response learning. We assessed effects of reactive- and proactive control on reaction time-, accuracy- and electroencephalographic time-frequency data. Behavioural results show distinct impairments of proactive- and reactive control in participants with Parkinson’s disease, when tested on their usual medication. Compared to healthy control participants, participants with Parkinson’s disease were impaired in their ability to adapt cognitive control proactively and were less effective to resolve conflict using reactive control. Successful reactive and proactive control in the healthy control group was accompanied by a reduced conflict effect between congruent and incongruent items in midline-frontal theta power. Our findings provide evidence for a general impairment of proactive control in Parkinson’s disease and highlight the importance of controlling for the effects of S-R learning when studying adaptive control. Evidence concerning reactive control was inconclusive, but we found that participants with Parkinson’s disease were less effective than healthy control participants in resolving conflict during the reactive control task.

## Teaser

Kricheldorff *et al*. compared performance in two adaptive control tasks in patients with Parkinson’s disease relative to healthy control participants. Successful reactive and proactive controls were accompanied by reduced conflict related theta power. Results demonstrate distinct impairments of proactive control in participants with Parkinson’s disease, tested on dopaminergic medications.

## Graphical Abstract

**Graphical Abstract:**
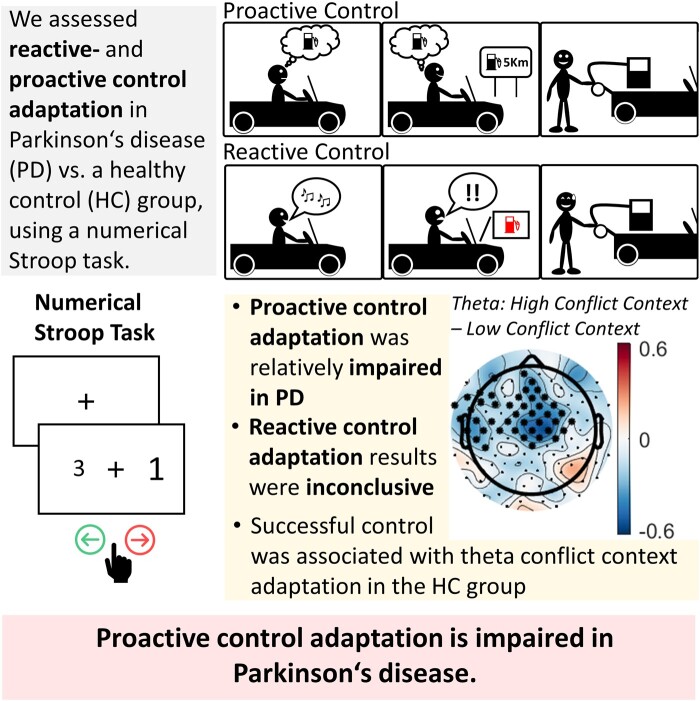

## Introduction

Parkinson's disease is a neurodegenerative disorder primarily diagnosed and characterized by symptoms causing impaired motor functioning. Parkinson’s disease also negatively affects multiple domains of cognition. Cognitive deficits associated with Parkinson’s disease are an even larger detriment to quality of life of patients with Parkinson’s disease than motor impairments.^1^ Individuals with Parkinson’s disease display deficits in inhibition,^2,3^ reinforcement learning^4^ and cognitive control.^5^ Impaired cognitive control can negatively affect other cognitive capacities that depend on cognitive control, such as adaptive control. Adaptive control is the ability to adjust cognitive control to a given context and is essential for successful goal-directed behaviour in dynamic environments.^6,7^ Evidence regarding impaired adaptive control in Parkinson’s disease is inconclusive.^8^ Therefore, gaining a better understanding of how Parkinson’s disease affects adaptive control is crucial.

Cognitive control describes the ability to regulate thoughts or behaviour to align with internal behavioural goals^9^ and is often measured using conflict tasks. For example, in the classical Stroop task^10^ participants have to name the colour of a displayed word. This is easy if colour and word match each other (congruent/no-conflict)—and difficult if colour and word do not match (conflict/incongruent). Cognitive control is required to resolve the ensuing conflict/interference and can be quantified by the strength of the conflict effect. The conflict effect measures the difference in reaction time (RT) or accuracy between trials containing conflicting/incongruent information versus trials containing non-conflicting/congruent information. The dual mechanisms of control framework^7,9^ posits two modes of cognitive control, proactive- and reactive control. Proactive control is effortful, sustained over time and already active before conflict is encountered. Reactive control is a ‘late-correction’ mechanism^9^ and only engaged after encountering conflict. Proactive control is considered costly and resource intensive, making reactive control the default option.^9^

### Measuring adaptive control

Adaptive control (also control learning or context-control learning) describes the adaptation of cognitive control resources to stable contexts repeatedly experienced over time. Proactive control adaptation is commonly measured by comparing the strength of the conflict effect between lists containing primarily conflicting [mostly incongruent (MI)] items to lists containing primarily non-conflicting [mostly congruent (MC)] items^11^—termed ‘list-wide proportion congruency effect’ (LWPCE). Cognitive control in MI contexts is sustained ‘globally’ over the whole list/block (i.e. a temporal context) in anticipation of upcoming conflicts and generalizes to new instances or items.^12^ This is reflected in a reduction in interference (i.e. the difference in reaction time or accuracy of conflict/incongruent items relative to non-conflict/congruent items) allowing participants to respond faster and more accurately in conflict trials. Moreover, RTs slowdown in non-conflict trials in the MI context compared to the MC context. In the MC context, control is seldom required and only applied reactively. Less control leads to more interference by the conflicting stimulus feature (slowing in RT and decrease in accuracy) on conflict/incongruent trials. Due to facilitation, RTs and error rates decrease on congruent trials. The congruency sequence effect (CSE) can also assess proactive control. The CSE is a local, transient measure of proactive control^11^ where adaptation in response to conflict in the previous trial is evaluated.^13^ LWPCE and CSE are similar in that conflict can be anticipated, and control resources are adapted proactively, but they differ in the scope of adaptation. LWPCE adaptation is global, sustained over the whole block, whereas in the CSE adaptation is local and confined to the next trial.

Reactive control adaptations can be measured by manipulating conflict proportions ‘locally’ at the item level—termed ‘item-specific proportion congruency effect’ (ISPCE).^11^ Specific items are presented more often containing conflicting information (MI), and others are presented more often containing non-conflicting information (MC). In contrast to the LWPCE, the temporal context across item types is balanced in terms of conflict presentation and conflict in the next item is unpredictable. Conflict adaptation can only occur bottom-up^12^ or reactively after encountering the item with its conflict predictive feature. Participants learn a ‘local’ feature-association, specific to a set of items. Reactive control is similarly estimated by calculating the difference in conflict effects between MC-Items and MI-Items.

Measurements of cognitive control adaptation have often been criticized because control adaptation is measured in the items used to induce cognitive control adaptation. This is problematic as the measured effect cannot only be explained by high-level cognitive control adaptation but also by low-level stimulus-response (S-R) learning (for a review, see Schmidt^14^), i.e. participants learn to respond to specific items but not context. To isolate adaptive control processes independently of S-R learning, Braem *et al*.^11^ recommend using one set of items to induce cognitive control adaptation where the proportion of congruent/incongruent presentation are manipulated (from here on referred to as ‘inducer-items’) and measure the effects in a second set of unbiased ‘diagnostic-items’ where the proportion of congruent/incongruent presentation is not manipulated i.e. the proportion of conflict and non-conflict trials presentation is balanced. For cognitive control adaptation to transfer, the diagnostic items must share a feature or context (e.g. the temporal context in the case of the LWPC manipulation) with the inducing items. The effect measured in the unbiased diagnostic items would reflect only high-level control adaptation independent of low-level S-R learning. In contrast, the effect in the inducing items may reflect a mix of both processes. Ideally, finding an effect in both the inducer- and diagnostic items would provide evidence for cognitive control adaptation. In contrast, the absence of the effect in the diagnostic items may indicate that low-level S-R learning processes best explain an effect in the inducer items.

### Adaptive control and Parkinson's disease

The literature on adaptive control in Parkinson's disease is ambiguous, showing both evidence in favour of intact- but also impaired adaptive control. Proactive control adaptation in Parkinson's disease has often been assessed using CSE manipulations. Participants with Parkinson's disease on their dopaminergic medication (DOPA-ON), in contrast to healthy control (HC) participants, have been reported to show comparable CSE modulations on RT^15^ and no CSE modulations^16,17^ on RT. Global proactive control adaptation, as measured by the LWPCE was reported absent in Parkinson's disease by one study.^8^ Successful adaptation of proactive control may also depend on dopamine replacement therapy. Duthoo *et al*.^18^ found the CSE to be impaired in participants with Parkinson's disease DOPA-ON, but not DOPA-OFF. In contrast, Ruitenberg *et al*.^19^ investigating global proactive control (LWPCE), found comparable conflict adaptation of movement speed in participants with Parkinson's disease both DOPA-ON and DOPA-OFF.

To our knowledge, reactive control in Parkinson's disease has only been assessed by Ruitenberg *et al*.^20^ using the ISPCE. Controlling for S-R learning effects, Ruitenberg *et al*.^20^ reported intact control adaptations in Parkinson's disease as compared to HC participants, independent of dopaminergic status.

The heterogeneous results, in conjunction with small sample sizes could suggest a potential reduction of local- and global proactive control in Parkinson's disease. Past studies did not distinguish between S-R learning and proactive control. Thus, it has yet to be established that participants with Parkinson's disease can acquire context-control rules independently of S-R learning.

### Electroencephalographical correlates of adaptive control

Electroencephalographical (EEG) correlates of cognitive- and adaptive control effects have often been investigated with event-related potentials (for an overview, see reference^18^), many of which have their spectral origin in the theta-band (4–8 Hz).^21^ Not only the phase-locked part of theta oscillations (i.e. the event-related potential), but to a larger degree also the non-phase locked part reflects conflict processing and is predictive of behaviour.^22^ Theta modulation over midline-frontal electrodes is thought to communicate the need for control.^21^ Reduced frontal theta in people with Parkinson's disease has been reported in a number of processes/tasks, such as the startle-response^23^ or interval timing.^24^ Moreover, people with Parkinson's disease exhibit reduced conflict-related midline-frontal theta activity relative to HC participants.^25^ Transient frontal theta activity has also been shown to increase in anticipation of a cognitive demanding task, indexing preparatory proactive control.^26^ Further, Chinn *et al*.^27^ found midline-frontal theta dynamics were modulated by proactive control adaptation (via LWPCE manipulation) with less frontal theta observed on conflict trials in the MI context relative to conflict trials in the MC context. Moreover, in local proactive control adaptation (CSE) less frontal theta was observed when a conflict trial was preceded by a conflict trial.^27,28^ Pastötter *et al*.^28^ traced the origin of the CSE theta cluster to the left cingulate gyrus and pre-supplementary motor area using multiple-source beamformer analysis. Regarding reactive control, Jiang *et al*.^29^ in a Stroop-like task with an ISPCE manipulation, found a posterior theta band cluster, with relatively increased power in incongruent trials on MI items. Thus, proactive control adaptation may be indexed by a smaller midline-frontal conflict theta effect, whereas reactive control adaptation may be characterized by a larger conflict theta effect, possibly with a more posterior distribution.

### The present study

Given the heterogeneous results investigating adaptive control in Parkinson's disease and methodological issues, the present study assesses if proactive and reactive control adaptation are affected in people with Parkinson's disease, using their usual medication regimen. In this experimental study, we used a numerical Stroop paradigm^30^ and separate items to induce the manipulation (inducer items) and measure its effect (diagnostic items). We expected reduced proactive control and intact reactive control in participants with Parkinson's disease in comparison to HC participants. Moreover, we explore midline-frontal theta activity as a possible correlate of potential behavioural deficits in reactive- and proactive control. We hypothesized reduced midline-frontal theta band activity to be associated with successful adaptive control and impairments to be indexed by a failure to adequately regulate midline-frontal conflict related theta activity. Moreover, we expected patterns in theta modulation to distinguish reactive from proactive control, with reactive control indexed by greater theta activity in incongruent trials under high conflict relative to low conflict and proactive control indexed by less theta activity in incongruent trials under high conflict relative to low conflict.

## Methods

### Ethics and registration

The study was approved by the local medical ethics committee (2020–133) in accordance with the declaration of Helsinki.^31^ The study design was pre-registered in the German clinical trial registry (DRKS00023020).

### Participants and general procedure

Between December 2020 and December 2021, we recruited 31 participants with Parkinson's disease and 34 healthy participants (HC) matched in age and gender. We did not perform a formal sample size calculation; instead, our sample size was determined by practical constraints. We considered how many participants were recruited in comparable studies assessing adaptive control in Parkinson's disease^8,15,17-20^ and which we could feasibly enrol within a year at our institution. Moreover, we used a short pilot experiment to determine the maximum number of items we could present to the participants in each experimental condition without overexerting them. All participants with Parkinson's disease fulfilled the movement disorder society (MDS)-diagnostic criteria for Parkinson's disease^32^ (see Table 1 for a summary of the clinical characteristics). Four of the HC participants were excluded because they did not use their right hand as instructed. One participant from the Parkinson's disease group was excluded because of inattentiveness as they frequently slept in during the task and infrequently responded only after verbal request (∼30% responded items). The final data set included 30 participants (22 men and 8 women) in the Parkinson's disease group (mean = 64 years, SD = 9.6 years) and 30 participants (18 men and 12 women) in the control group (mean = 59.4 years, SD = 6.8 years). Participants with Parkinson's disease were older than the HC participants (*t*(55) = 2.25, *P* = 0.03), but did not differ significantly in terms of gender (*X*² (1) = 0.675, *P* = 0.41).

**Table 1:** Clinical data of the Parkinson's disease group

Characteristic	Count/Mean (SD)
Affected side	
Left	N = 13
Right	N = 13
Both	N = 4
Disease subtype	
Akinetic-rigid	N = 18
Tremor-dominant	N = 4
Equivalent type	N = 8
Disease duration (years)	5.3 (3.7)
LEDD (mg)	557 (259)
Hoehn and Yahr	2.0 (0.5)
UPDRS	12.0 (7.0)

Mean values and standard deviations shown in parenthesis. LEDD, levodopa daily equivalent dose.

We included participants fulfilling the following criteria (i) no co-morbid neurological or psychiatric problems; (ii) right-handedness; (iii) normal or corrected-to-normal vision; (iv) mini-mental state examination (MMSE) >25 (mean_PD_ = 29.2, SD_PD_ = 1.1; mean_HC_ = 29.1, SD_HC_ = 1.0, *t*(58) = 0.12, *P* = 0.9). UPDRS-III ratings were recorded for all participants with Parkinson's disease (mean = 12.0, SD = 7.0). Moreover, we recorded the formal education in years (mean_PD_ = 16.2 years, SD_PD_ = 3.0 years; mean_HC_ = 16.0 years, SD_HC_ = 3.2 years, *t*(58) = 0.25, *P* = 0.8).

The experiment took place at the out-patient clinic of the Evangelisches Krankenhaus Oldenburg. After being informed about the purpose of the experiment, participants gave their written informed consent and were screened with the MMSE and reported their education level. MDS-UPDRS-III ratings were recorded for all participants with Parkinson's disease. Subsequently, participants performed the experiment and EEG was recorded. In total, task and preparation lasted for about 2.5 h.

### Experimental paradigm

**Figure 1:**
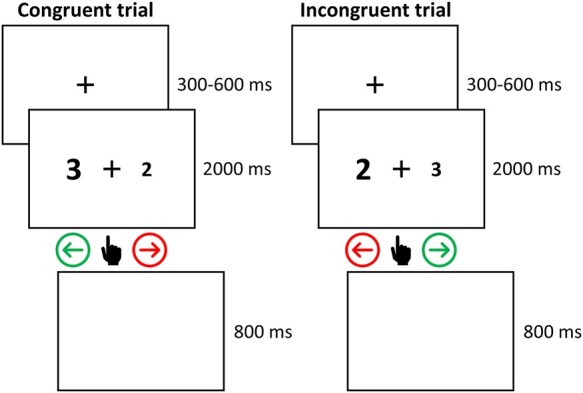
**Task description.** Overview of task timing and illustrations for an incongruent and congruent trial.

Participants performed a numerical Stroop task with LWPCE and ISPCE manipulations, presented in OpenSesame.^33^ Two numbers were displayed. One number was numerically larger than the other and one number was displayed physically larger than the other. The task required participants to select the numerically larger number on a keyboard by pressing the left arrow key indicating the number on the left and the right arrow key indicating the number on the right. Participants were instructed to use their right hand to respond. Congruency-conflict effects were introduced by manipulating the physical size of the number pairs. We selected the numerical Stroop task to: (i) have sufficiently many items available for inducer and diagnostic item sets and (ii) reduce task demands by avoiding participants having to learn multiple response options. During each trial participants saw a fixation cross for 300–600 ms (uniformly varied), followed by the two numbers presented on screen until participants indicated a response. Participants had up to 2000 ms to make a response and the RT and accuracy was recorded for each trial. A blank screen was displayed for 800 ms before the next trial started (see Fig. 1).

Participants completed eight blocks with 134 trials each. Four blocks contained LWPCE items and four blocks contained ISPCE items. Per block, ∼70% of the items presented were inducer items (N = 94) and ∼30% (N = 40) were diagnostic items. Diagnostic items contained equal proportions of congruent and incongruent items. Inducer items contained ∼80% incongruent items in the MI condition and 80% congruent items in the MC condition. In the LWPCE manipulation, participants were presented with two blocks containing more- (MI—70% incongruent- 30% congruent trials) and two blocks containing fewer (MC—30% incongruent- 70% congruent trials) incongruent items. In each of the four ISPCE blocks, equal amounts of congruent and incongruent items were presented. For the ISPCE, we manipulated the proportion of congruency by the numerical size of the number pairs—larger number pairs (numbers >5) versus smaller number pairs (numbers <5; see Supplementary Table 1 for a list of all items) for half of the items. For example, two large number pairs were randomly selected as inducer items, and the remaining two large number items were used as diagnostic items. We used the same items as used by Dadon and Henik.^34^ For half of the participants (per experimental group) the large number items contained MC comparisons and the small items MI comparisons and vice versa for the other half of the participants. Before performing the task, participants completed 32 trials with feedback, without the LWPCE or ISPCE manipulations, to familiarize themselves with the task.

Block presentation order was counterbalanced. Half of the participants would either start with the ISPCE blocks or the LWPCE blocks. Within the LWPCE blocks, the presentation order was also counterbalanced, with half of the participants (per group) starting with the MI blocks and the other half with MC blocks. Items were presented in a pseudo-randomized order. Pseudo-randomization was performed with custom-written Python scripts, using a mix of random shuffling and backtracking^35^ to achieve a random presentation of the criteria we defined (for the criteria, see Supplementary Information 1).

### EEG recording and preprocessing

EEG was continuously recorded (actiChamp plus by Brain Products^36^) with a sampling rate of 1000 Hz, from 128 active Ag/AgCl electrodes. Impedances were kept below 10 kΩ. Preprocessing was performed in Matlab using the EEGLAB toolbox.^37^ Data were down-sampled to 250 Hz, 0.1 Hz high-pass filtered and detrended over the whole recording period for each channel separately. Noisy channels were excluded (on average 4.9(SD = 4.1) channels per participant) via the pop_clean_rawdata() function with the following criteria: FlatlineCriterion = 5, ChannelCriterion = 0.8, and LineNoiseCriterion = 5. Afterwards, data were re-referenced to an average reference. Line-noise was removed using EEGLABs pop_cleanline() function. Noisy data segments were excluded in a automatized manner with artefact subspace reconstruction^38^ using the pop_clean_rawdata() function with the ‘Burstcriterion’ parameter set to 80. A relatively high threshold was selected to remove only excessively noisy data segments. Subsequently, we used independent component analysis (Infomax algorithm) to identify and remove components reflecting eye blinks and larger muscle artefacts. Data were epoched stimulus-locked [−1.2 s, 2 s] and response-locked [−2 s, 1.2 s]. To decrease the effects of volume conduction, we performed a surface-Laplacian transformation using the CSD toolbox.^39^

After preprocessing in the group with Parkinson's disease, we excluded one participant due to insufficient data quality (excessive movement during recording), and two participants whose data contained too few trials (<40) in some conditions of the ISPCE task to perform the regression analysis. The same was true for two participants in the LWPCE condition. The final data set in the LWPCE and ISPCE analyses of the Parkinson's disease group consisted of 26 participants. In the HC group, one participant was excluded due to a technical problem during recording and one participant had too few trials (<40) for the ISPCE regression analysis. The final data set of the HC group consisted of 29 participants for LWPCE analysis and 28 participants in the ISPCE analysis.

### EEG data analysis

Response-locked and stimulus-locked data sets were frequency transformed via fast Fourier transform and convolved with the FFTs of a series of Morlet wavelets. We used 20 wavelets logarithmically spaced from 2 to 30 Hz. Wavelet cycles were logarithmically spaced from three to ten, increasing by frequency. This allowed better band specific resolution for the higher frequencies and better time resolution for lower frequencies. After wavelet convolution, data were down-sampled to 125 Hz for further analysis. Due to a short fixation window (300–360 ms), baseline correction was performed over the whole trial period.^40^ A baseline period covering 0–1000 ms (or −1000–0 ms for the response-locked data) was used for decibel conversion. This approach allowed us to identify transient changes in oscillatory activity.

### Statistical analysis

#### Behaviour

Analyses of the behavioural data were performed in R (R-4.1.3).^41^ Data visualizations were created using the ggplot2 package^42^ and ggdist.^43^

Statistical analyses of the behavioural data were performed using Bayesian mixed effect models with the brms package.^44^ For the RT analysis (performed on correctly answered trials) due to right-skewed distribution of RT data, we used a shifted log-normal likelihood function. Trials with RTs shorter than 200 ms were excluded (20 trials or 0.03% of the data). The ISPCE and LWPCE were analysed in separate models. We used contrast coded dummy variables to calculate the main effects for Congruency (congruent–incongruent), Block/Item Type (MC–MI) and their interaction for each group (Parkinson's disease–HC) separately (see Supplementary Table 2). Moreover, we included random intercepts for the participants and items and separate intercepts per group for the shift parameter of the model.

We fitted separate inducer models for the LWPCE- and ISPCE data. For the inducer models, we used weakly informative priors derived at by prior predictive simulations yielding plausible RT distributions of the data (see Supplementary Equation 1). To maximize the utility of the data, we used information from the inducer items to inform the prior parameter space of the diagnostic models (see Supplementary Information 2 for a detailed description).

To evaluate the parameter estimates in milliseconds, we used the posterior distribution to calculate the estimated marginal means for each effect (MC congruent, MC incongruent, MI congruent, and MI incongruent) and group. Next, we calculated the ‘Conflict effect’ (congruent–incongruent) for each of the two conflict conditions (MC and MI). Adaptive control was operationalized as the difference between the two conflict effects and improved performance in conflict/incongruent trials (incongruent trials MI–incongruent trials MC). To assess group differences, we compare the improvement of performance in conflict/incongruent trials between both groups using the posterior marginal mean distributions of the model. The error data were analysed in a similar manner but due to low error-rate without clear results (see Supplementary Information 3, Supplementary Equation 2 and Supplementary Table 3 for a summary).

To evaluate how well a particular parameter predicted the data, we calculated Bayes inclusion factors (BIF) across matched models using bridge sampling^45^ for each experimental group separately. For example, to evaluate the contribution of Congruency in the HC group, we did not consider the contribution of the factors in the Parkinson's disease group. The BIF of Congruency reflects the likelihood of factor Congruency ($H_{A}$) over the averaged likelihood of the null model and the model containing the factor Block ($H_{0}$). BIFs and Bayes Factors smaller than 1 suggest evidence favouring the null model, whereas BIFs and Bayes Factors larger than 1 would signify evidence favouring the alternative. We qualify evidential strength provided by the BIF by using criteria suggested by Jeffreys.^46^ BIFs smaller than 3 and larger than 1/3 provide anecdotal evidence for either hypothesis. BIFs between 3 and 10 (or 1/3 and 1/10) provide moderate evidence, between 10 and 30 (1/10 and 1/30) strong evidence and anything larger than 30 (or <1/30) is classified as substantial evidence in support of the hypothesis in question.

For the Markov chain Monte Carlo sampling, we ran four chains with 2000 warm-up iterations and 10 000 iterations for each chain to sample from the posterior distribution. We assessed model convergence by confirming that the potential scale reduction factor R̀ for all parameters was near 1 and <1.1 and visual inspection of the chain trace plots to verify if the individual chains are similar and vary around similar parameter estimates. Chain trace plots visualize how well individual chains mix and are commonly used as a graphical diagnostic method to assess convergence (for more details, see for example^47^). Model fit was assessed comparing the actual data with simulated data from the model’s posterior predictive distribution (see Supplementary Figs 2 and 3).

#### EEG

After decibel conversion of the EEG data, we performed a regression analysis using the ordinary least-squared solution for each participant, electrode, frequency and time point using functions provided by the LIMO toolbox.^48^ We used effect coded contrasts with separate models for the LWPCE and ISPCE data. For each participant we had contrasts for Congruency, Block/Item and their interaction. As the number of diagnostic items was too small to be analysed individually we collapsed inducer and diagnostic items to fit the models. To investigate the interaction effect between Block/Item and Congruency, marginal mean effects were calculated for each participant. With the marginal mean effects, we calculated the difference in Congruency (congruent–incongruent) for the conflict effect in each block/item (MC and MI). Adaptive control (ISPCE or LWPCE) was then analysed by comparing the difference in conflict effects between contexts where proportionally more—or less conflict is expected (MI–MC). We averaged the data over the theta-band (4–8 Hz) to investigate the contribution of conflict-related theta activity. To correct for multiple comparisons, we used non-parametric cluster-based permutation test statistics^49^ in FieldTrip^50^ using dependent *t*-tests, a cluster-alpha of 0.05 and an alpha of 0.05, with 10 000 permutations.

## Results

### Behavioural results

**Figure 2:**
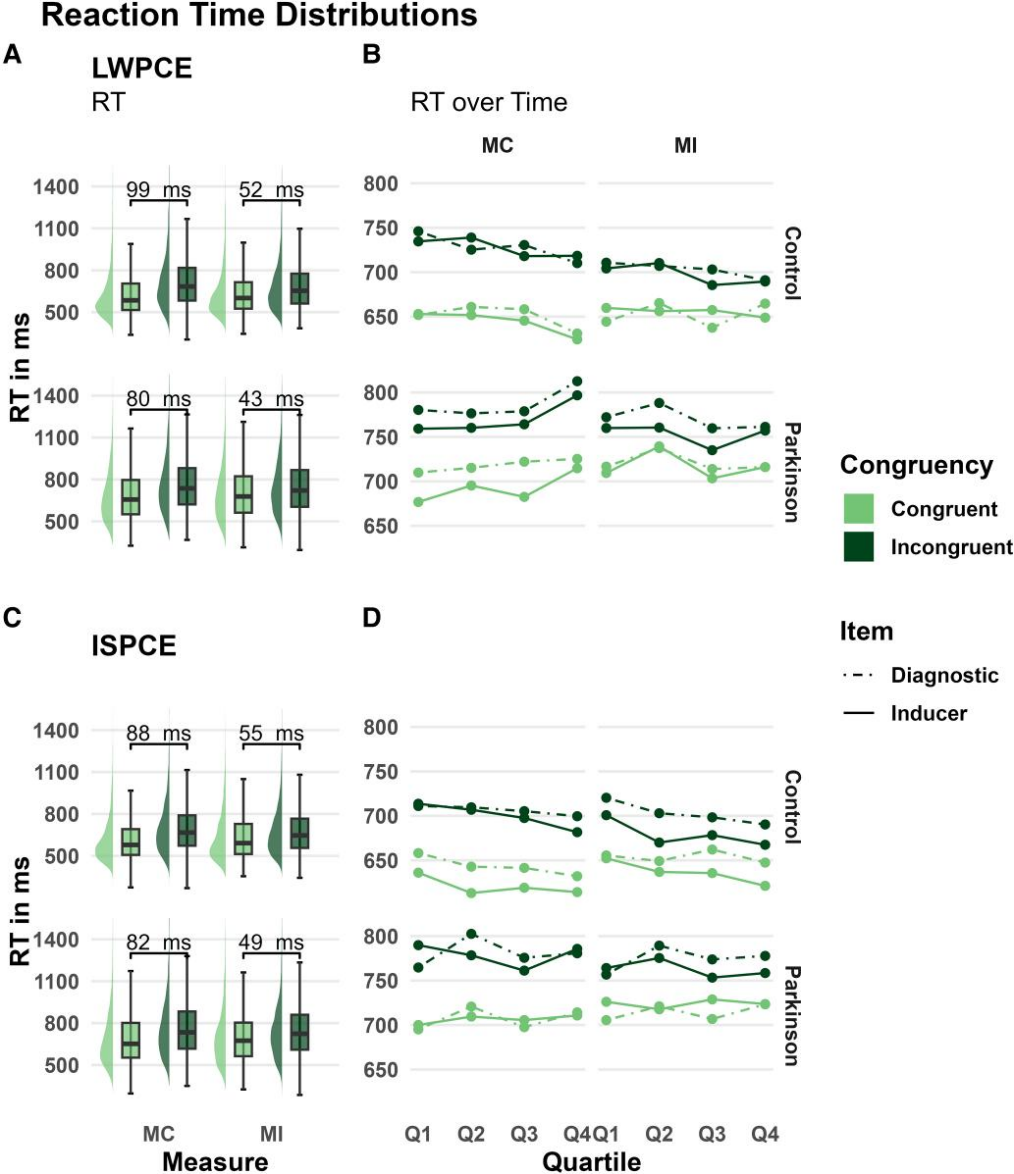
**Reaction time (RT) distributions and consistency of diagnostic and inducer item reaction times over time of the list-wide proportion congruency effect.** Effects are displayed separately for the LWPCE- (**A**, **B**) and item-specific proportion congruency effect—ISPCE data (**C**, **D**). The top row contains data for the HC participants (N = 30) and bottom row of the participants with Parkinson's disease (N = 30). (**A**, **C**) depict the RT distribution of all items (diagnostic and inducer) by congruence for the high conflict [mostly incongruent (MI)] and low conflict [mostly congruent (MC)] proportion manipulation. Nodes at the bottom show the average difference in RT between congruent and incongruent items by proportion manipulation. (**B**, **D**) display the time course of RT averaged over quartiles by congruency (congruent and incongruent) and item type (inducer and diagnostic items). The two columns distinguish the high- (MI) and low (MC) conflict proportion manipulation.

We found anecdotal evidence that participants with Parkinson's disease were slower (747 ms) compared to the HC participants (673 ms) in overall RT performance, *t*(58) = 2.11, *P* = 0.039, *BF_10_* = 1.66 (see Fig. 2). We found strong evidence that participants with Parkinson's disease performed on average more errors (3.1%) than the HC participants (1.5%), *t*(58) = −3.1, *P* = 0.004, BF_10_ = 11.53. Nonetheless, overall accuracy in the task was high across groups and we found only very limited evidence for the use of adaptive control in the analysis of the error data (summarized in Supplementary Table 3).

**Figure 3:**
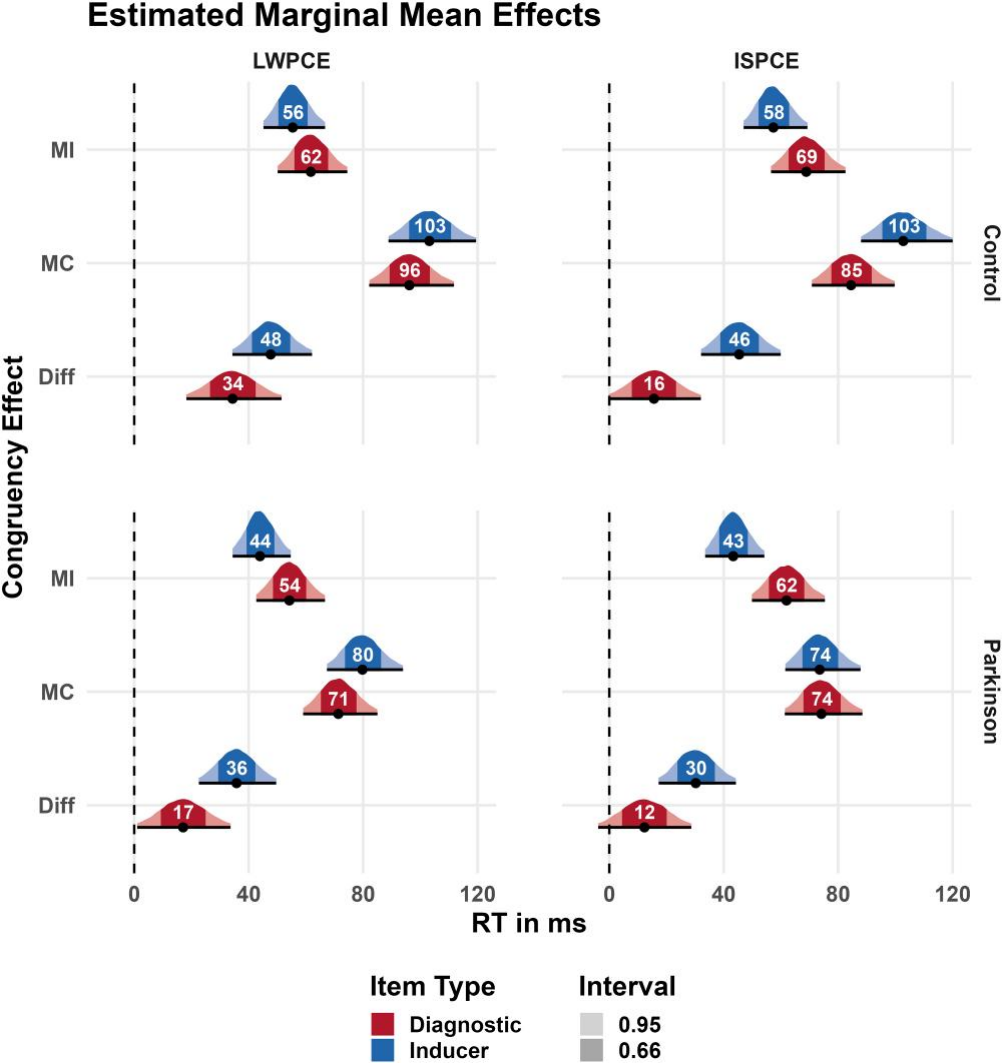
**Summary of the shifted log-normal regression analysis results.** Displayed are the estimated marginal mean posterior distributions for the conflict effects (incongruent–congruent) in the high conflict condition [mostly incongruent (MI)], the low conflict condition [mostly congruent (MC)], and their difference. Results are organized row-wise by Control- (N = 30) and Parkinson group (N = 30) and column-wise by effect [list-wide proportion congruency effect (LWPCE) and item-specific proportion congruency effect (ISPCE)]. The shaded areas of the posterior distribution correspond to the 66% and 95% credible intervals.

Results of the shifted-log normal analysis are depicted in Table 2 and Fig. 3. The RT analysis of the inducer items of the LWPCE manipulation showed an interaction effect, with reduced conflict RT in the MI versus MC condition, for both the HC participants (*m* = −47.8 ms, 95% CI = [−62.1 ms, −34.3 ms], BIF > 1000) and the participants with Parkinson's disease (*m* = −35.9 ms, 95% CI = [−49.6, −22.6 ms], BIF > 1000). The effect in the HC group was explained largely by a reduction in RT to incongruent items in the MI condition (*m* = −29.9 ms, 95% CI = [−40.5 ms, −19.8 ms]). The Parkinson's disease group showed a similar pattern with a reduction in RT to incongruent items in the MI condition (*m* = −26.2 ms, 95% CI = [−36.8 ms, −16.2 ms]). In the analysis of the diagnostic items, we also found substantial evidence for an interaction effect in the HC group, (*m* = −34.5 ms, 95% CI = [−51.5 ms, −18.2 ms], BIF = 518.32), with the effect being driven by a reduction in RT to incongruent items in the MI condition (*m* = −27.8 ms, 95% CI = [−39.9 ms, −16.3 ms]). The interaction effect in the diagnostic items was smaller in the Parkinson's disease sample and provided anecdotal evidence (*m* = −17.2 ms, 95% CI = [−33.7 ms, −1.1 ms, BIF = 1.74]), for an interaction effect. Contrasts revealed possibly no reduction in RT to incongruent items in the MI condition (*m* = −7 ms, 95% CI = [−18.5 ms, 4.3 ms]) with the credible interval being compatible with null. Moreover, the difference between groups in posterior probability in RT reduction to incongruent items in the MI condition indicates that HC participants had a larger RT reduction (*m* = 20.8 ms, 95% CI = [37.1 ms, 4.8 ms]). Thus, it appears that relative to HC participants, participants with Parkinson's disease were impaired in their ability to extend proactive control to diagnostic items. This can be seen in Fig. 2B as the RTs of the diagnostics items track the RTs of the inducer items closely in the HC participants but not the participants with Parkinson's disease.

**Table 2:** Results of the shifted log-normal regression analysis

Parameter	Parkinson's disease		HC	
	Mean estimate and 95% CI in ms	BIF_10_	Mean estimate and 95% CI in ms	BIF_10_
LWPCE—Inducer Items				
Congruency	62.1 [52.9, 72.3]	>1000	79.5 [68.7, 91.4]	>1000
Block PC	8.3 [1.8, 14.9]	0.19	6.0 [−0.5, 12.6]	0.04
Interaction	−35.9 [−49.6, −22.6]	>1000	−47.8 [−62.1, −34.3]	>1000
LWPCE—Diagnostic Items				
Congruency	62.9 [53.9, 72.7	>1000	79.1 [68.9, 90.4]	>1000
Block PC	−1.5 [−8.4, 5.3]	0.35	10.6 [3.7, 17.7]	14.80
Interaction	−17.2 [−33.4, −1.1]	1.74	−34.5 [−51.5, −18.2]	518.32
ISPCE—Inducer Items				
Congruency	58.6 [49.5, 69.0]	>1000	80.3 [69.2, 93.0]	>1000
Item PC	−1.1 [−7.4, 5.5]	0.03	20.9 [14.1, 28.1]	>1000
Interaction	−30.4 [−44.2, −17.3]	>1000	−45.5 [−59.9, −32.1]	>1000
ISPCE—Diagnostic Items				
Congruency	68.2 [58.3, 79.1]	>1000	76.9 [66.2, 88.5]	>1000
Item PC	−6.2 [−13.3, 0.9]	3.00	17.0 [9.8, 24.4]	235.78
Interaction	−12.3 [−28.7, 3.9]	0.68	−15.6 [−32.0, 0.2]	0.53

Mean estimates and 95% credible intervals are provided in milliseconds. The factor congruency reflects the difference between incongruent and congruent items, the factor Block/Item proportion congruency (PC)the relative difference between MC and MI blocks/items and the interaction reflects the difference in conflict effects (incongruent–congruent) in the MI blocks/items relative to the MC blocks/items. BIF exceeding 1000 or smaller 0.001 are abbreviated for the purpose of making the table legible.

We explored if participant-specific LWPCEs for the Parkinson's disease group were associated with motor status of the participants (see Supplementary Information 4 for a description). However, there was no statistically significant correlation between MDS UPDRS motor scores and LWPCEs in the inducer- (*r* = 0.35, *P* = 0.067), and diagnostic items (*r* = 0.28, *P* = 0.14). Other clinical variables such as disease duration, Hoehn and Yahr stage, affected body side and disease subtype did not show a significant association either (for a summary of these results, see Supplementary Fig. 1).

Lastly, we explored in a sensitivity analysis whether the results were affected by the inclusion of post-error trials. Results did show very small deviations in the estimated effect sizes and no changes in the qualitative conclusions drawn from the reported analysis (see Supplementary Table 4).

The analysis of the inducer items of the ISPCE analysis revealed again substantial evidence for an interaction effect in both the HC- (*m* = −45.5 ms, 95% CI = [−59.9 ms, −32.1 ms], BIF > 1000) and Parkinson's disease participant (*m* = −30.4 ms, 95% CI = [−44.2 ms, −17.3 ms], BIF > 1000) groups. Conflict processing on incongruent items in the MI condition was improved in HC-(*m* = −43.6 ms, 95% CI = [−55.3 ms, −32.8 ms]) and participants with Parkinson's disease (*m* = −14.2 ms, 95% CI = [−24.3 ms, −4.6 ms]). However, comparing the differences in posterior probability between groups showed that HC participants had a larger reduction with a mean of 24.8 ms [95% CI = 1.6 ms, 8.6 ms]. Thus, while both groups show a sizable ISPCE effect on RTs in the inducer items, the HC participants appeared to be more effective in regulating responses to conflict than the participants with Parkinson's disease. Further analysis of the diagnostic items provided anecdotal evidence against an ISPCE, favouring the null hypothesis in the HC- (*m* = −15.6 ms, 95% CI = [−32.0 ms, 0.2 ms], BIF = 0.53) and group with Parkinson's disease (*m* = 12.3 ms, 95% CI = [−28.7 ms, 3.9 ms], BIF = 0.68). Hence, we found no evidence that either group could extend reactive control beyond the inducer items.

#### EEG results

**Figure 4:**
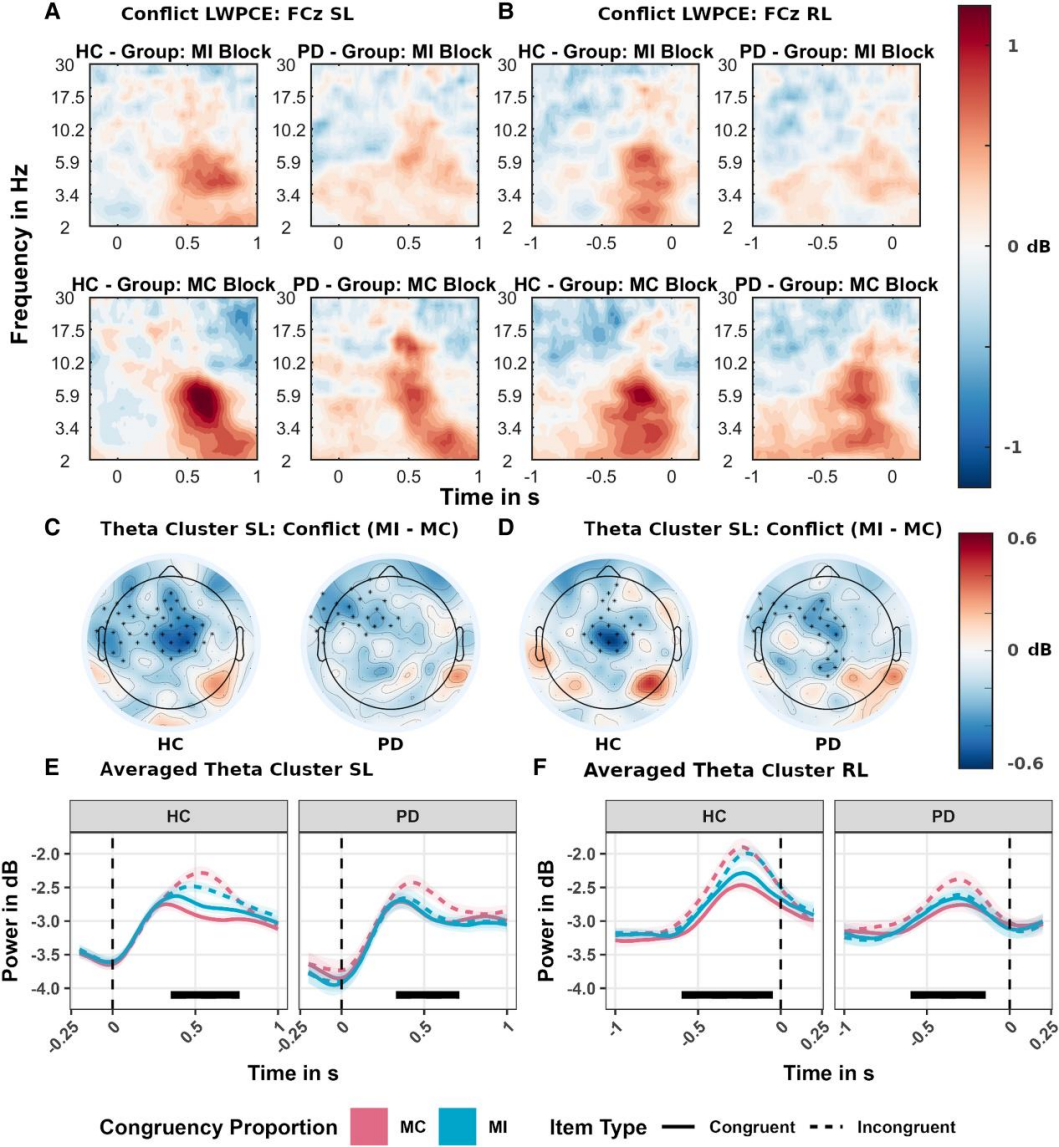
**Estimated marginal mean effects of the time-frequency regression analysis for the list-wide proportion congruency effect (LWPCE) manipulation**. Data on the left (**A**, **C**, **E**) are plotted in reference to stimulus onset (SL), and data on the right (**B**, **D**, **F**) are referenced to the response (RL). (**A**, **B**) show the time-frequency results at the FCz electrode (**A**, **B**) for the conflict effect (incongruent–congruent) by conflict proportion manipulation [mostly incongruent (MI) and mostly congruent (MC)] and HC (N = 29) and Parkinson's disease (N = 26) group). (**C**, **D**) depict the results of the theta band (4–8 Hz) cluster-permutation analysis on the difference in conflict effects between conflict proportion manipulation (MI–MC). Significant clusters are marked with an asterisk symbol. The underlying colour gradient depicts the averaged difference in conflict theta activity in decibel (dB) over the period when the significant cluster was detected. (**E**, **F**) depict the estimated marginal mean effects in theta activity, averaged over electrodes of the significant cluster. Shaded areas depict the standard errors (SE) at each sample. Marked in black is the period when the significant cluster was detected: for the HC group post-stimulus onset (0.35 s to 0.77 s; *P* = 0.0004) and prior to response (−0.6 s to −0.05 s; *P* = 0.002); for the Parkinson's disease group post stimulus onset (0.33 s to 0.71 s; *P* = 0.0088) and prior to response (−0.6 s to −0.14 s; *P* = 0.0012).

We assessed whether conflict related power in the theta band was reduced in MI- versus MC contexts. Figure 4A and B plot the conflict effect (incongruent–congruent) at the FCz channel for the LWPCE. Based on visual inspection, power appears to be larger, at frequencies between 4–8 Hz, on incongruent trials than congruent trials (condition specific plots can be found in the Supplementary Figs. 4–7). The conflict effect is larger in the MC condition, than the MI condition particularly in the HC group as shown by a significant negative cluster (MI conflict effect–MC conflict effect) after stimulus presentation (0.35 s to 0.77 s; *P* = 0.0004), largely over midline-frontal to frontal-left channels. A negative cluster could also be observed prior to response execution (−0.6 s to −0.05 s; *P* = 0.002), with a more midline-frontal distribution. In the Parkinson's disease group, we similarly find a significant negative cluster after stimulus presentation (0.33 s to 0.71 s; *P* = 0.0088) and prior to response execution (−0.6 s to −0.14 s; *P* = 0.0012). Both clusters show a frontal left distribution (see Fig. 4). Thus, both groups show a reduction in midline-frontal conflict related theta activity when more conflict is expected. Figure 4E and F further show the modulation by estimated marginal mean condition averaged over the electrodes identified in the significant cluster.

**Figure 5:**
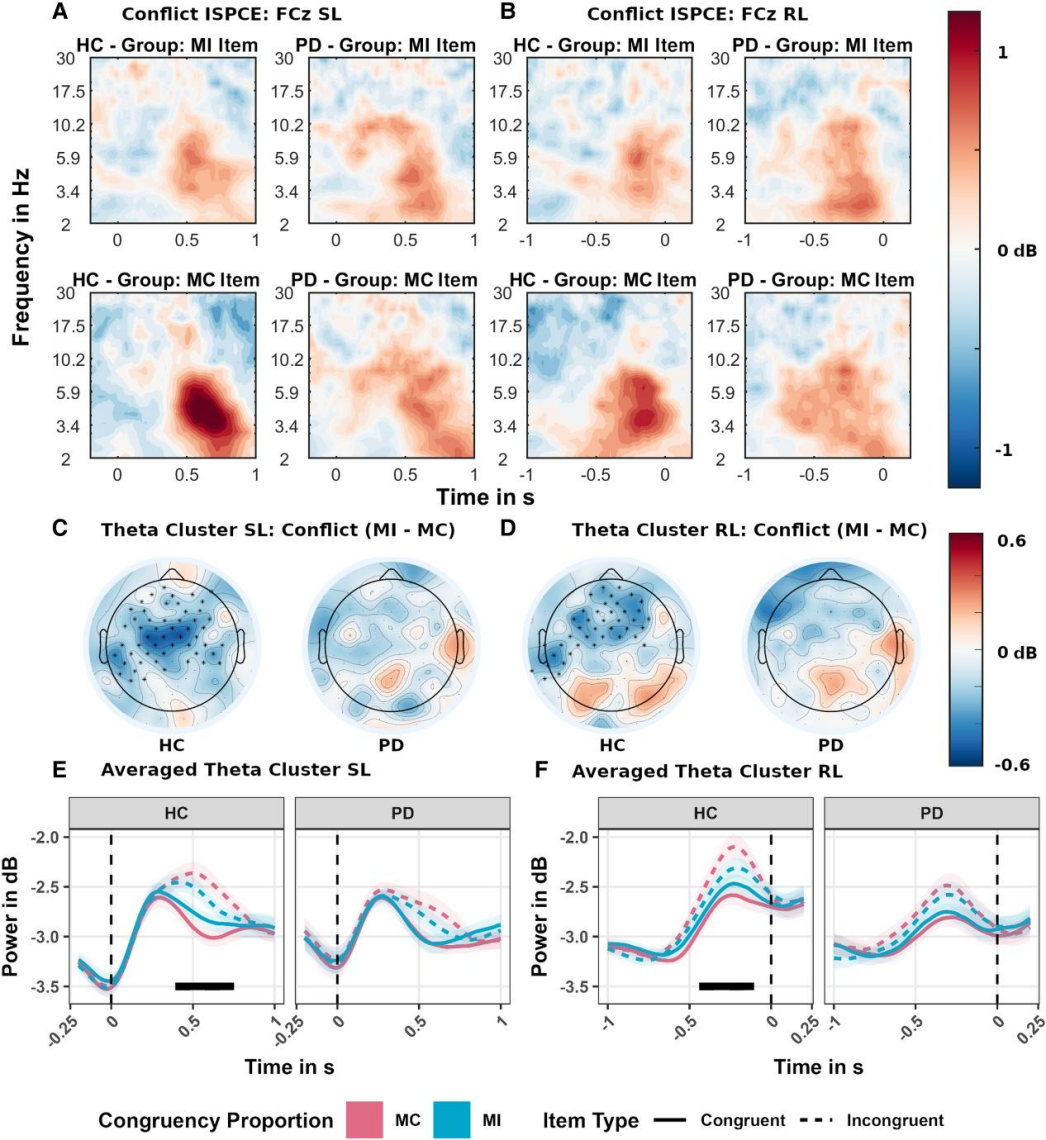
**Estimated marginal mean effects of the time-frequency regression analysis for the item-specific proportion congruency effect (ISPCE) manipulation**. Data on the left (**A**, **C**, **E**) are plotted in reference to stimulus onset (SL), and data on the right (**B**, **D**, **F**) are referenced to the response (RL). (**A**, **B**) show the time-frequency results at channel FCz (**A**, **B**) for the conflict effect (incongruent–congruent) by conflict proportion manipulation [mostly incongruent (MI) and mostly congruent (MC)] and HC (N = 28) and Parkinson's disease (N = 26) group. (**C**, **D**) depict the results of the theta band (4–8 Hz) cluster-permutation analysis on the difference in conflict effects between conflict proportion manipulation (MI–MC). Significant clusters are marked with an asterisk symbol. The underlying colour gradient depicts the averaged difference in conflict theta activity over the period when the significant cluster was detected. In the Parkinson's disease, panels where no significant difference was detected, we display the activity in decibel (dB) between 0.3 and 0.7 s and −0.6 s and −0.2 s for comparison. (**E**, **F**) depict the estimated marginal mean effects in theta activity, averaged over electrodes of the significant cluster in the HC group. Shaded areas depict the standard errors (SE) at each sample. Marked in black is the period when the significant cluster was detected: for the HC group, post-stimulus onset (0.39 s to 0.75 s; *P* = 0.0017) and prior to response (−0.44 s to −0.1 s; *P* = 0.0039).

For the ISPCE (Fig. 5A and B), the theta conflict effect at the FCz electrode is larger in the MC condition than the MI condition only in the HC group as shown by a negative cluster over midline-frontal electrodes (see Fig. 5C and D) both post-stimulus onset (0.39 s to 0.75 s; *P* = 0.0017) and prior to response (−0.44 s to −0.1 s; *P* = 0.0039). Thus, when using reactive control only the HC participant showed reduced theta conflict power in items where high conflict was expected (MI).

## Discussion

The present study investigated whether adaptive control is impaired in medicated participants with Parkinson's disease. We created a task to investigate both proactive control- and reactive control adaptation and compared performance in participants with Parkinson's disease to HC participants. We distinguished between manipulation inducing items and unbiased diagnostic items to avoid confounding S-R learning. Our results show an impairment in the acquisition of general context-control associations for proactive control adaptation in Parkinson's disease. Further, our results showed evidence of reactive control adaptation in both groups, but participants with Parkinson's disease displayed a reduced ability to suppress conflict compared to HC participants.

### Interpretation of adaptive control in Parkinson's disease

We observed a specific impairment to form general temporal context-control associations by participants with Parkinson's disease in the task requiring proactive control. The initial analysis of the inducer RT data showed strong evidence for the ability of both participants with Parkinson's disease and HC participants to use proactive control. Both groups displayed reduced RTs on incongruent trials in high conflict temporal context (MI), compared to low-conflict temporal context (MI). However, in the assessment of the unbiased diagnostic items we observed strong evidence that the HC-, but not the participants with Parkinson's disease, could regulate proactive control. This qualitative difference between both groups in the diagnostic data demonstrates that participants with Parkinson's disease are impaired in the ability to learn context-control associations necessary for proactive control. Effects observed in the inducer items of the Parkinson's disease group are more likely to be explained by S-R learning.

Our findings on the inducer items are in line with Ruitenberg *et al*.^19^ who observed significant conflict adaptation effects in participants with Parkinson's disease both ON- and OFF- their dopaminergic medication performing a Stroop task. We extend results by Ruitenberg *et al*.^19^ by showing that this adaptation does not transfer to general context dependent adaptation and suggest that the results are better explained by S-R learning. In contrast Bonnin *et al*.^8^ have reported proactive, global control adaptations to be impaired in participants with Parkinson's disease (DOPA-ON). However, Bonnin *et al*.^8^ never directly compared proactive control in participants with Parkinson's disease to HC participants and interpreted a non-significant interaction effect in participants with Parkinson's disease as evidence of impaired conflict modulation. Similar to Bonnin *et al*.^8^ our *post hoc* analysis did not show a strong association with UPDRS scores. Thus, proactive control deficits, may not be solely explained by disease progression or severity.

Behavioural results suggest that participants with Parkinson's disease could also be impaired in reactive control adaptation. In the analysis of the inducer items, participants with Parkinson's disease and HC participants displayed strong evidence for an interaction effect indicative of reactive control. However, further inspection revealed that HC participants were more effective in reducing RT conflict cost on incongruent trials in items associated with high conflict. The analysis of the diagnostic items showed no evidence for reactive control adaptation in both groups. There are two plausible explanations for the result of the diagnostic items: (i) the manipulation worked as intended, and neither the HC participants nor the participants with Parkinson's disease could exert reactive control; (ii) the manipulation failed, and reactive control did not transfer to the diagnostic items. We believe that the latter is the case. The numerical sizes that we contrived to be ‘small’ (1–4) and ‘large’ (5–8) in this experiment are likely not a feature of magnitude that people naturally encode and that could be linked to control learning. Pilot data of a younger HC group not reported here that should have the capacity of reactive control adaptation similarly did not show evidence of reactive control in the diagnostic items. However, since conflict modulation was already reduced in the inducer items in participants with Parkinson's disease, it is unlikely that we would observe a larger adaptation effect in the diagnostic items than in the inducer items. Nonetheless, since we cannot assess the contribution of S-R learning to reactive control adaptation without assessing the diagnostic items, these results are a prompt for future inquiries rather than evidence of impaired reactive control in Parkinson's disease.

The reactive control effect in the inducer items observed in the participants with Parkinson's disease resemble results by Ruitenberg *et al*.^20^ who found no impairment in reactive control irrespective of participants being tested ON- or OFF their dopaminergic medication. However, because compared to the HC participants the participants with Parkinson's disease exhibited a reduced capacity to improve the processing of conflict trials we cannot exclude the possibility of impaired reactive control adaptation. Ruitenberg *et al*.^20^ reported the difference in Stroop effects irrespective of the nature of the cost effect (incongruent/congruent items). It would be interesting to learn to what degree the effects in their task depend on improved conflict processing.

### Interpretation of electrophysiological adaptive control effects

Midline-frontal theta modulation was associated with successful proactive- and reactive conflict adaptation in the HC group. Previous studies of proactive control on the CSE (Pastötter *et al*.^28^) and the LWPCE (Chinn *et al*.^27^) also report reduced conflict theta during conflict processing. However, in our task participants with Parkinson's disease also showed evidence of modulation of midline-frontal theta despite their inability to form general context-control associations. Therefore, the presence of conflict theta modulations during conflict processing alone does not reflect intact proactive control adaptation.

We observed a similar modulation of conflict midline-frontal theta for reactive control adaptation in the HC participants but not the participants with Parkinson's disease. In conjunction to their reduced ability to improve on conflict trials, this suggests that reduced conflict theta in high conflict trials may be a necessary condition for effective reactive control. Our results show the opposite pattern of theta modulation described by Jiang *et al*.^29^ However, the theta cluster Jiang *et al*.^29^ identified was located over midline-posterior electrodes, not midline-frontal electrodes. Moreover, the study was performed in young and healthy participants. Thus, it is conceivable that aging effects may also affect the patterns of control related theta identified in both studies.

The results of both experimental manipulations suggest that successful adjustment of control to context is associated with a reduction in midline-frontal theta in response to conflicting stimuli. This reduction in theta power could reflect more efficient use of conflict resolution resources in control adaptation. When the context predicts a high probability of conflict, less control resources are required—or vice-versa when conflict expectation is low more control resources are required to resolve encountered conflict.

### Explanations for deficits observed in Parkinson’s disease

Two possible interpretations of the deficit observed in Parkinson's disease come to mind. The first is the dopamine-overdose hypothesis.^51-53^ Parkinson's disease is associated with a loss in midbrain dopamine cells. Due to differential neurodegenerative progression in dorsal striatal circuits and ventral striatal circuits,^54^ dopamine replacement therapy can have divergent effects on cognition. Cognitive functions reliant on impaired dorsal striatal circuits may profit, whereas functions relying on intact ventral striatal circuitry may be impaired. Brain regions associated with conflict monitoring are thought to rely more on the spared ventral striatal circuits.^55^ Thus, it would be reasonable to expect impaired performance in medicated participants with Parkinson's disease as tested here. Future research should assess if performance in unmedicated patients with Parkinson's disease is not impaired in a diagnostic test set.

Another interpretation, independent of dopaminergic medication, could be that participants can learn simple stimulus-response associations but cannot perform sustained top-down control, informed by learned priors. Perugini *et al*.,^56^ observed that participants with Parkinson's disease did not incorporate prior information in their decisions in a probabilistic perceptual decision-making task. Participants learned that a particular response was more likely within a given temporal context or item feature and were presented with easy (certain) and difficult (uncertain) decisions. Participants with Parkinson's disease did not use this information under conditions with high uncertainty, where it would be most relevant and responded stimulus-driven. The failure to use prior information is believed to reflect basal ganglia impairment,^57,58^ as it was observed irrespective of dopaminergic status (ON/OFF)^57^ and in participants with dopamine unresponsive focal dystonia, a disorder with impaired basal ganglia function but unaffected frontal dopamine circuits.^57^ This could also be interpreted as a failure to engage in control adaptation and continued reliance on uninformed stimulus-driven responding. The ‘monochromatic’ task (temporal context predicts response) used^56^ shares features of proactive control adaptation, whereas the ‘dichromatic’ task (item feature colour predicts response) resembles reactive control adaptations. The studies differ that in the Perugini *et al*. studies^56,57^ participants could predict a concrete response, whereas in our study they could predict conflict. Nonetheless, it is striking how in conflict adaptation and informed perceptual decision-making, participants with Parkinson's disease appear to struggle to translate learned probabilistic stimulus information into concrete behavioural adaptations.

### Strength and limitations

To our knowledge, the present study is the first to evaluate both proactive control and reactive control in Parkinson's disease while controlling for S-R learning. We improved on previous work by having a relatively large sample size (30 participants per group), using appropriate statistical models to assess effects on reaction time distributions, and analysing EEG frequency correlates of adaptive control in Parkinson's disease. Nonetheless, results need to be viewed in light of some limitations. Our conclusions regarding reactive control are limited as our diagnostic manipulation was unsuccessful. The feature of the items that we manipulated may have been too abstract. However, a failure to identify an ISPCE in the diagnostic or transfer items is not uncommon. For example, Bejjani *et al*.^59^ did not find an ISPCE in the diagnostic items. Further, concerning reactive control, the proportion congruency manipulation after the removal of participants was no longer counterbalanced and equal across groups. After we excluded participants who had used both hands to respond, for 18 participants in the HC group, the larger number items contained MI comparisons. In contrast, in the group with Parkinson's disease, it was only 14 participants. Thus, we cannot exclude the possibility that this may have impacted the magnitude of the effect between both groups. Furthermore, we did not screen participants for ON/OFF fluctuations during the recording. While no ON/OFF fluctuations of participants in the Parkinson's disease group were noted by the experimenters, this could have negatively influenced the performance of some participants in the Parkinson's disease group. Further, our conclusions of the EEG analysis are limited due to our choice of a short fixation interval, to maximize the amount of trials/power to detect an effect. Using the whole trial duration as a baseline allowed us to identify differences in transient modulations of midline-frontal theta, but not sustained changes. Lastly, while our study provides evidence of the state of adaptive control in participants with Parkinson's disease on their dopaminergic medication, it is yet to be conclusively determined to what degree adaptive control is impacted by disease and medication.^20,55^

### Conclusion

We demonstrated distinct impairments of proactive control and possibly reduced reactive control in participants with Parkinson's disease, when tested on their usual medication. Participants with Parkinson's disease appear to be capable to adjust cognitive control to items directly associated with specific cognitive control demands but cannot form general proactive context-control associations. These adaptations cannot easily be reinterpreted in terms of intact reactive control or caused by S-R learning. Participants with Parkinson's disease, in contrast to HC participants, were less effective to regulate cognitive control in the reactive control task in items with high cognitive control demands. A distinguishing feature may have been the salience in conflict signalling the need of control adaptation. Moreover, results of our elderly control sample highlight that successful reactive and proactive control adaptations may be accompanied by reduced conflict related midline-frontal theta power. Results concerning reactive control and Parkinson's disease should be further validated in future studies with successful transfer to diagnostic items. Moreover, it would be interesting to learn to what degree midline-frontal theta generalizes to unbiased context-control associations. Further studies are required to further dissociate to what degree adaptive control deficits can be explained in terms of Parkinson's disease and medication.

## Supplementary Material

fcad327_Supplementary_Data

## Data Availability

Due to containing information that could compromise the privacy of research participants and missing consent the data cannot be made publicly available. The experiment and code used for the analysis and experiment can be found at: https://osf.io/bsn8v/
