# Supplementary material for: Impaired proactive cognitive control in Parkinson’s disease
Source: Brain Commun. 2023 Nov 30;5(6):fcad327. doi: 10.1093/braincomms/fcad327 (PMC10733811; doi:10.1093/braincomms/fcad327)
Supplement: fcad327_Supplementary_Data [file fcad327_supplementary_data.pdf]

Supplementary Material – Impaired proactive cognitive control in Parkinson's disease

Items		
Numerical Distance	Small Number Pairs	Large Number Pairs
1	1-2, 3-4	6-7, 8-9
2	1-3, 2-4	6-8, 7-9

Supplementary Table 1 List of items used in the numerical Stroop task. We used items with a numerical distance 1 and 2, also previously used by Dadon and Henik[1]. Items were balanced in terms of numerical presentation and overall presentation. For the ISPCE manipulation, either small or large number pairs were manipulated respectively.

Criteria were (1) diagnostic items should not be presented one after each other (in order to have a balanced presentation of diagnostics throughout the block), (2) the same item should not be presented more than twice in a row, (3) inducer items should not be presented more than four times in a row, (4) the same correct response side (left or right) should not be presented on more than three consecutive trials and (5) the same congruence should not be presented more than four times in a row. Item presentation orders were randomized for each participant separately according to these rules.

Supplementary Information 1 Criteria for pseudo-randomization.

	$C_{CongruencyHC}$	$C_{ProportionHC}$	$C_{InteractionHC}$	$C_{CongruencyPD}$	$C_{ProportionPD}$	$C_{InteractionPD}$
Incongruent_MI_HC	-0.5	-0.5	0.5	0	0	0
Incongruent_MC_HC	-0.5	0.5	-0.5	0	0	0
Congruent_MC_HC	0.5	0.5	0.5	0	0	0
Congruent_MI_HC	0.5	-0.5	-0.5	0	0	0
Incongruent_MI_PD	0	0	0	-0.5	-0.5	0.5
Incongruent_MC_PD	0	0	0	-0.5	0.5	-0.5
Congruent_MC_PD	0	0	0	0.5	0.5	0.5
Congruent_MI_PD	0	0	0	0.5	-0.5	-0.5

Supplementary Table 2 Contrast Matrix

$$\log(RT_{nm} - \exp(ndt)) \sim Normal(\mu_{nm}, \sigma)$$

$$\mu_{nm} = \alpha + u_{subj[n],1} + u_{Item[m],2} + \beta_1 C_{Congruency_{PD}} + \beta_2 C_{Proportion_{PD}} + \beta_3 C_{Interaction_{PD}} + \beta_4 C_{Congruency_{HC}} + \beta_5 C_{Proportion_{HC}} + \beta_6 C_{Interaction_{HC}}$$

$$ndt = \beta_7 PDGroup + \beta_8 HCGroup$$

$$\sigma \sim Normal_+(0, 0.5)$$

$$\alpha \sim Normal_+(6.5, 0.5)$$

$$\beta_1 \sim Normal(0, 0.3)$$

$$\beta_2 \sim Normal(0, 0.3)$$

$$\beta_3 \sim Normal(0, 0.3)$$

$$\beta_4 \sim Normal(0, 0.3)$$

$$\beta_5 \sim Normal(0, 0.3)$$

$$\beta_6 \sim Normal(0, 0.3)$$

$$u_1 \sim Normal(0, 0.3)$$

$$u_2 \sim Normal(0, 0.3)$$

$$\beta_7 \sim Normal(5.3, 0.5)$$

$$\beta_8 \sim Normal(5.3, 0.5)$$

Supplementary Equation 1 Shifted log-normal regression model and priors.

For the diagnostic model, we used the posterior distribution of the inducer model to construct informed priors for the effects not expected to differ between conditions (shift parameter, sigma, Congruency, Block/Item, and the random intercepts). We used a Gaussian distribution, with mu equal to the mean of the inducer posterior and sigma defined as the larger absolute difference between the posterior mean and the two 95% credible interval borders of the inducer posterior. For the interaction effects, we used regularized priors with a Gaussian distribution centered around zero, with sigma defined as the larger absolute difference between zero and the two 95% credible interval borders of the posterior distribution of the inducer model.

Supplementary Information 2 Informed prior distributions of the diagnostic models.

$$Error_{nm} \sim Bernoulli(\theta_{nm})$$

$$\eta_{nm} = \left(\frac{\exp(\theta_{nm})}{1 + \exp(\theta_{nm})}\right)$$

$$\eta_{nm} = \alpha + u_{subj[n],1} + u_{Item[m],2} + \beta_1 C_{Congruency_{PD}} + \beta_2 C_{Proportion_{PD}} + \beta_3 C_{Interaction_{PD}} + \beta_4 C_{Congruency_{HC}} + \beta_5 C_{Proportion_{HC}} + \beta_6 C_{Interaction_{HC}}$$

$$\alpha \sim Normal(-1.3, 1.5)$$

$$\beta_1 \sim Normal(0, 1.5)$$

$$\beta_2 \sim Normal(0, 1.5)$$

$$\beta_3 \sim Normal(0, 1.5)$$

$$\beta_4 \sim Normal(0, 1.5)$$

$$\beta_5 \sim Normal(0, 1.5)$$

$$\beta_6 \sim Normal(0, 1.5)$$

$$u_1 \sim Normal(0, 1.5)$$

$$u_2 \sim Normal(0, 1.5)$$

Supplementary Equation 2 Logistic regression model and priors

For the analysis of the error data, we performed a logistic regression (Bernoulli distribution with logit-link function) and used the same approach to fit inducer and diagnostic models as outlined for the shifted-log normal model. Priors were weakly informed so that lower error rates had a higher probability, as typically, participants never make more than 10% errors during these kinds of tasks (see supplement Supplementary Equation 2). Log odds of the estimated marginal mean effects were transformed into probabilities for a more meaningful interpretation.

Supplementary Information 3 Description of the logistic regression.

**Parameter**	**PD**		**HC**	
	Mean Error & 95%CI	*BIF_{10}*	*Mean Error & 95%CI*	*BIF_{10}*
		**LWPCE – Inducer Items**		
Congruency	2.0[1.2, 3.2]	>1000	2.6[1.6, 4.1]	>1000
Block PC	0.7[0.2, 1.4]	1.04	1.4[0.7, 2.4]	0.75
Interaction	-1.2[-2.4, -0.2]	2.10	-3.1[-5.2, -1.6]	5.88
		**LWPCE – Diagnostic Items**		
Congruency	3.0[1.9, 4.5]	>1000	2.6[1.6, 4.0]	>1000
Block PC	0.6[0.0, 1.4]	4.36	0.6[-0.1, 1.4]	0.71
Interaction	-0.7[-2.1, 0.6]	1.74	-2.0[-3.7, -0.6]	2.95
		**ISPCE – Inducer Items**		
Congruency	1.1[0.6, 2.0]	>1000	2.2[1.1, 4.0]	>1000
Item PC	0.5[0.2, 1.1]	<0.001	0.4[-0.3, 1.3]	<0.001
Interaction	-1.7[-3.1, -0.8]	2.10	-1.4[-3.3, -0.1]	0.001
		**ISPCE – Diagnostic Items**		
Congruency	1.5[0.8, 2.5]	>1000	2.8[1.5, 4.7]	>1000
Item PC	0.1[-0.3, 0.6]	<0.001	0.5[-0.2, 1.4]	<0.001
Interaction	-0.2[-1.1, 0.6]	<0.001	-2.1[-4.3, -0.6]	0.009

Supplementary Table 3 Results of the logistic regression analysis. Mean error estimates and 95% credible intervals are provided in percent. The factor congruency reflects the difference between incongruent and congruent items, and the factor Block/Item PC is the relative difference between MC and MI blocks/items, and the interaction reflects the difference in conflict effects (incongruent - congruent) in the MI blocks/items relative to the MC blocks/items. BIF exceeding 1000 or smaller than 0.001 are abbreviated for the purpose of making the table legible.

After identifying a deficit in the LWPCE manipulation in the PD group, we were interested in the association between proactive control and the motor status of the participants. We fitted the inducer and diagnostic model (with the previously informed priors) to the data of the participants with PD alone. Again, we assessed the effects of Block, Congruency, and the interaction. In contrast to the previous models, we included random slopes for the main and interaction effects. The resulting model gave us participant-specific posterior distributions. We calculated proactive control as the difference in Stroop effects between the two conflict conditions (MC and MI), with a posterior distribution of the differences for each participant. We summarized proactive control as the mean of the resulting posterior distribution and calculated the Pearson correlation with the participant-associated MDS-UPDRS motor scores. To evaluate statistical significance in this analysis, we calculated p-values with a significance level alpha of 0.05.

Supplementary Information 4 Exploratory Analysis LWPCE

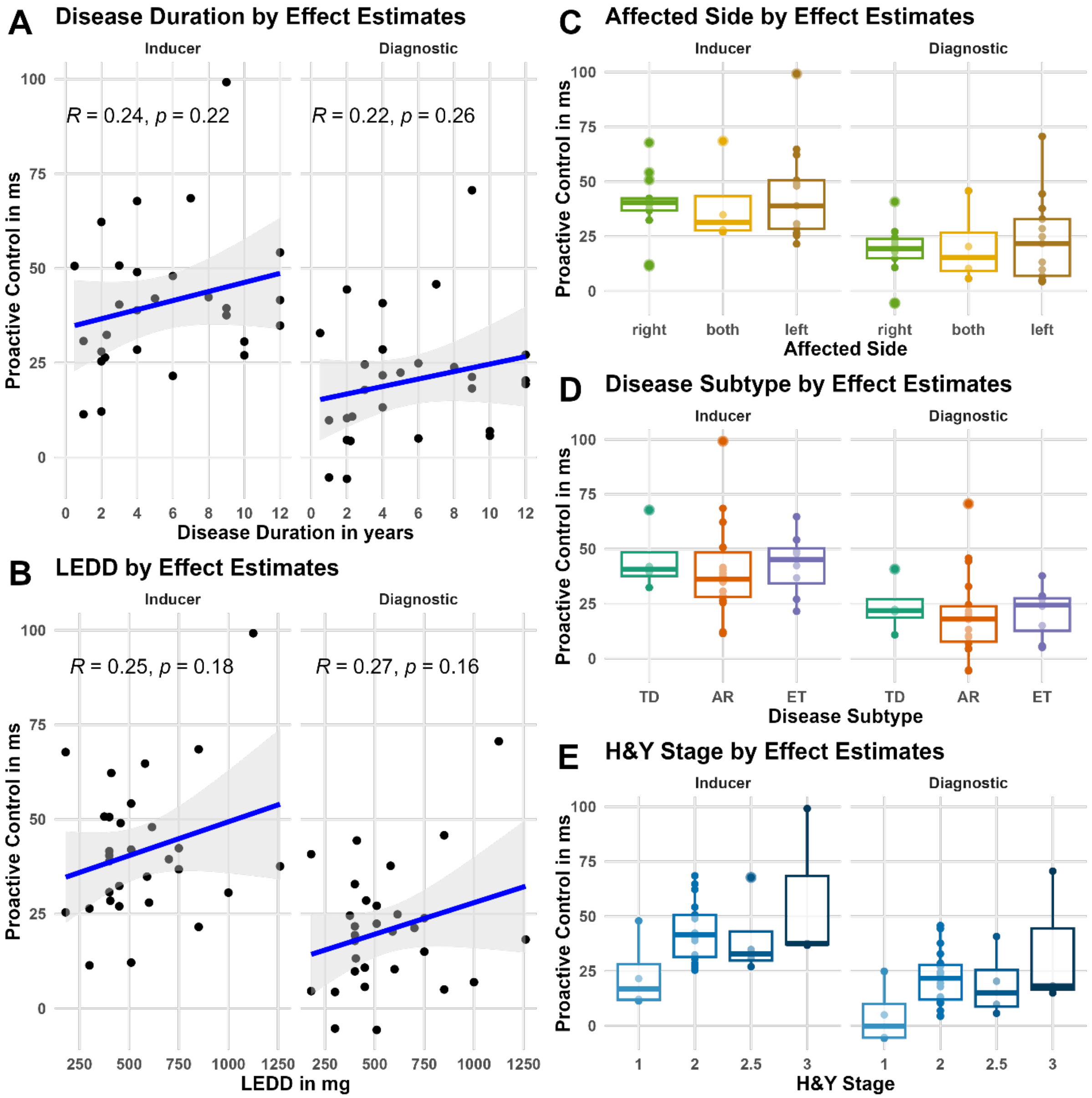

Supplementary Figure 1 Associations between clinical variables and individual participant-specific estimated marginal mean effects (EMME) (N = 30) for the diagnostic and inducer items, respectively. Panels A and B show the association between disease duration and EMME and levodopa daily equivalence dose (LEDD) and EMME. Test statistics are depicted at the top of each panel. Panel C shows EMME by affected body side (left, right, or equally affected). Calculating the Welch t-test, there was no significant difference in EMME between the left and right body side in the inducer items (t(21.4) = 0.7; p = 0.49) and diagnostic items (t(20.4)) = 0.89, p = 0.39). Panel D shows EMME by disease subtype (TD = tremordominant type; AR = akineto-rigid type; ET = equivalent type). Calculating the Welch t-test, there was no significant difference in EMME between AR and ET side in the inducer items (t(19.6) = -0.43; p = 0.67) and diagnostic items (t(21.5)) = -0.22, p = 0.83). Panel E shows EMME by Hoehn and Yahr Stage.

**Parameter**	**PD**		**HC**	
	Mean RT & 95%CI with post-error trials	*Mean RT & 95%CI without post-error trials*	*Mean RT & 95%CI with post-error trials*	*Mean RT & 95%CI without post-error trials*
		**LWPCE – Inducer Items**		
Congruency	62.1[52.9, 72.3]	62.3[52.6, 73.4]	79.5[68.7, 91.4]	83.9[71.1, 97.8]
Block PC	8.3[1.8, 14.9]	8.1[1.5, 14.9]	6.0[-0.5, 12.6]	5.5[-1.1, 12.2]
Interaction	-35.9[-49.6, -22.6]	-36.6[-51.1, -23.1]	-47.8[-62.1, -34.3]	-49.2[-65.0, -35.9]
		**LWPCE – Diagnostic Items**		
Congruency	62.9[53.9,72.7]	63.4[53.5, 74.4]	79.1[68.9, 90.4]	83.0[71.4, 95.9]
Block PC	-1.5[-8.4, 5.3]	-2.2[-9.1, 4.8]	10.6[3.7, 17.7]	10.4[3.5, 17.6]
Interaction	-17.2[-33.4, -1.1]	-19.9[-36.4, -3.4]	-34.5[-51.5, -18.2]	-39.3[-56.6, -22.7]
		**ISPCE – Inducer Items**		
Congruency	58.6[49.5, 69.0]	57.2[48.1, 67.5]	80.3[69.2, 93.0]	80.9[69.8, 93.9]
Item PC	-1.1[-7.4, 5.5]	-1.1[-7.6, 5.5]	20.9[14.1, 28.1]	20.8[14.2, 27.9]
Interaction	-30.4[-44.2, -17.3]	-31.9[-45.7, -18.7]	-45.5[-59.9, -32.1]	-46.4[-60.5, -33.1]
		**ISPCE – Diagnostic Items**		
Congruency	68.2[58.3, 79.1]	69.5[59.5, 80.4]	76.9[66.2, 88.5]	76.6[66.1, 87.9]
Item PC	-6.2[-13.3, 0.9]	-6.2[-13.4, 0.8]	17.0[9.8, 24.4]	16.9[9.8, 24.3]
Interaction	-12.3[-28.7, 3.9]	-10.2[-26.8, 6.0]	-15.6[-32.0, 0.2]	-17.6[-33.8, -1.7]

Supplementary Table 4 Results of the shifted log-normal regression sensitivity analysis. Mean estimates and 95 percent credible intervals are provided in milliseconds. For each group, the left-hand column presents the parameter estimates conditioned on data, including post-error trials, and the right-hand column shows the parameter estimates conditioned on the data excluding post-error trials. The factor congruency reflects the difference between incongruent and congruent items. The factor Block/Item PC is the relative difference between MC and MI blocks/items, and the interaction reflects the difference in conflict effects (incongruent - congruent) in the MI blocks/items relative to the MC blocks/items. BIF exceeding 1000 or smaller than 0.001 are abbreviated to make the table legible.Supplementary Table 1 List of items used in the numerical Stroop task. We used items with a numerical distance 1 and 2, also previously

used by Dadon and Henik[1]. Items were balanced in terms of numerical presentation and overall presentation. For the ISPCE manipulation, either small or large number pairs were manipulated respectively.

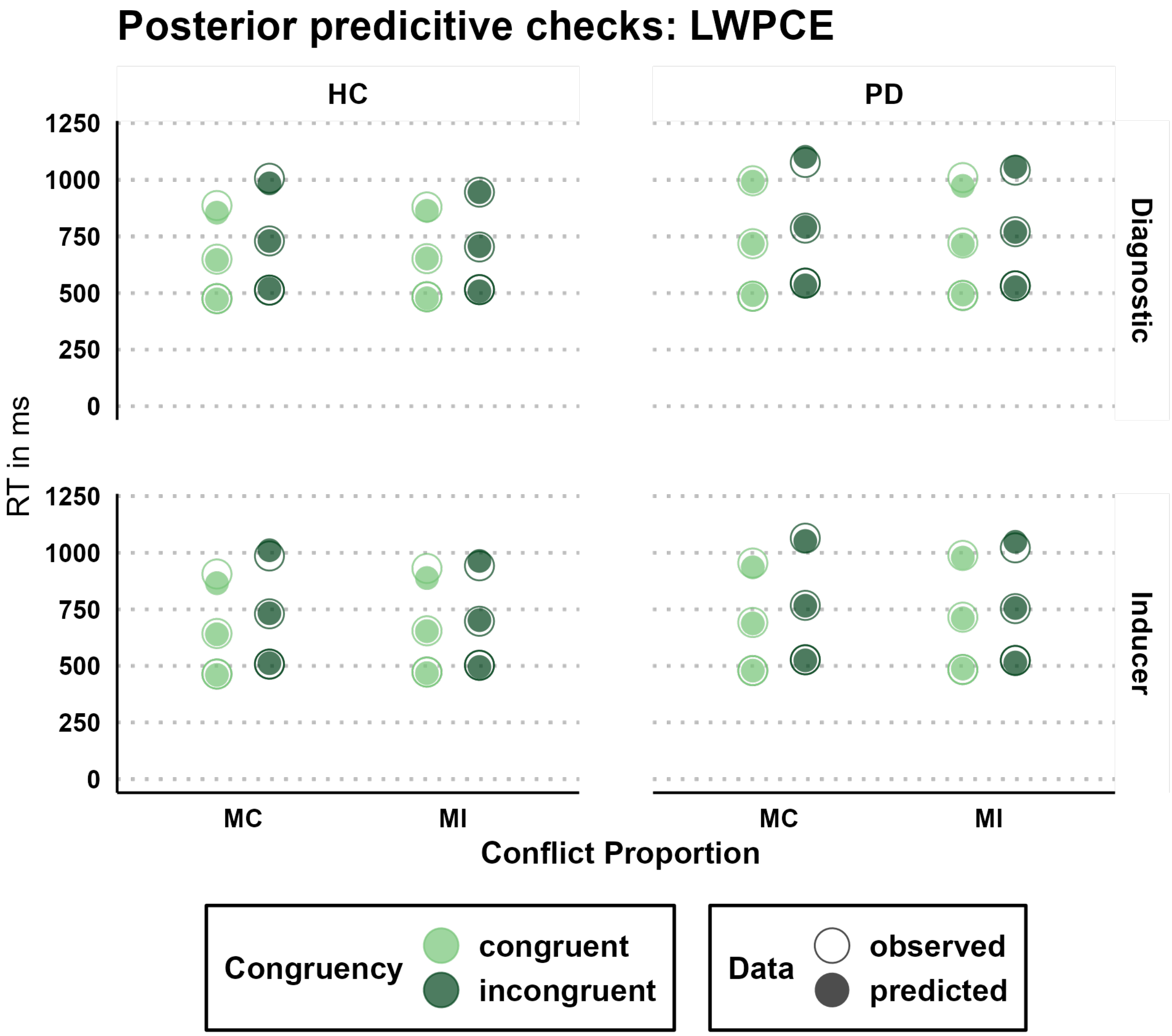

Supplementary Figure 2 Posterior predictive checks of reaction times for the analysis of the LWPCE data. Hollow dots depict the observed data, and full dots represent the posterior predictions of the shifted log-normal model. Light green colored dots show congruent items and dark green dots show reaction times to incongruent items. Rows distinguish reaction times to inducer and diagnostic items. Within each panel, the upper dot reflects the mean of the upper 90th percentiles of the reaction time distribution. The middle dot reflects the average of the reaction time distribution, and the lower dot is the mean of the 10th percentile of the reaction time distribution.

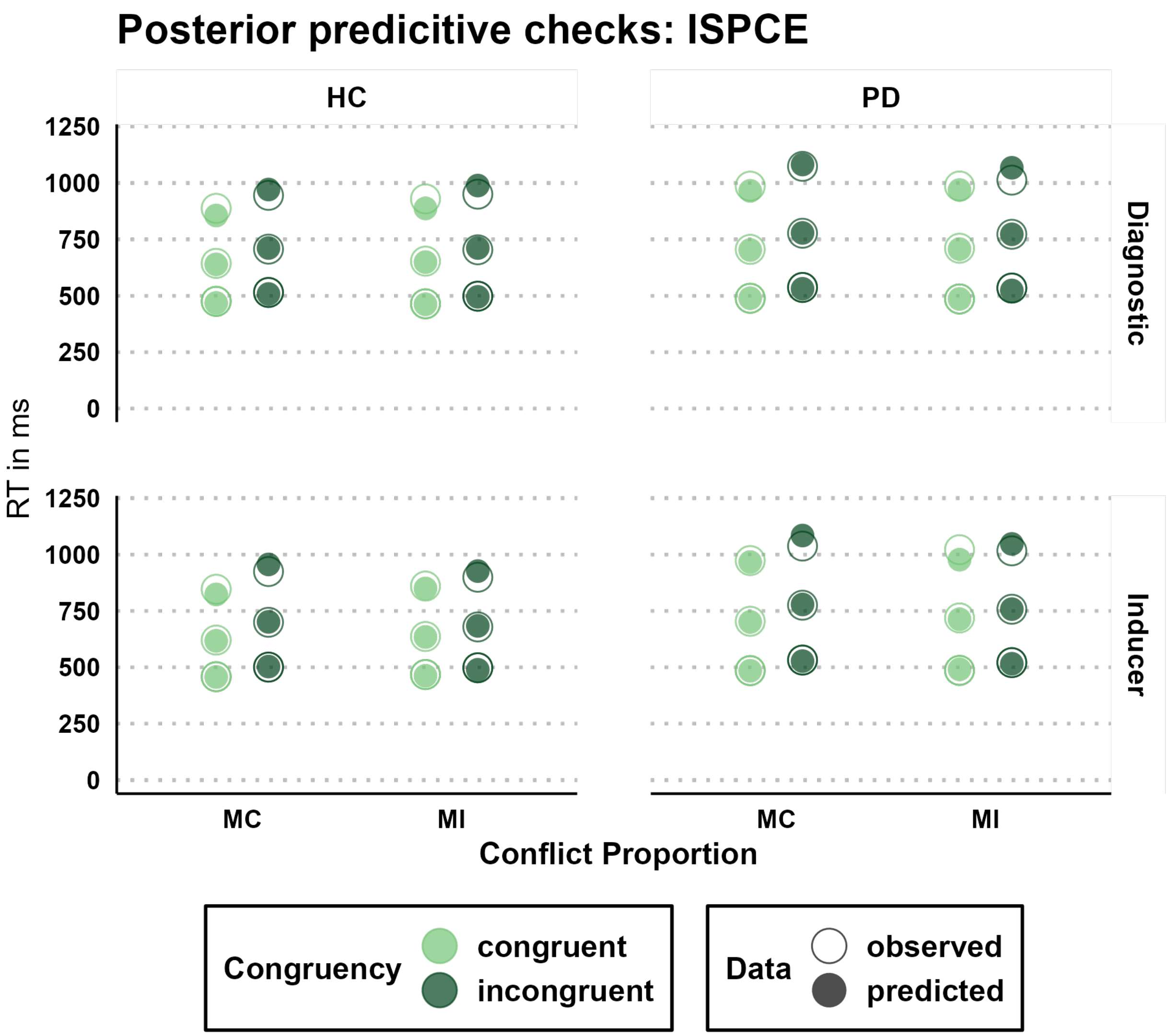

Supplementary Figure 3 Posterior predictive checks of reaction times for the analysis of the ISPCE data. Hollow dots depict the observed data, and full dots are the posterior predictions of the shifted log-normal model. Light green colored dots show congruent items and dark green dots show reaction times to incongruent items. Rows distinguish reaction times to inducer and diagnostic items. Within each panel, the upper dot reflects the mean of the upper 90th percentiles of the reaction time distribution. The middle dot reflects the average of the reaction time distribution, and the lower dot the mean of is the 10th percentile of the reaction time distribution.

1. Condition Specific Time Frequency Results

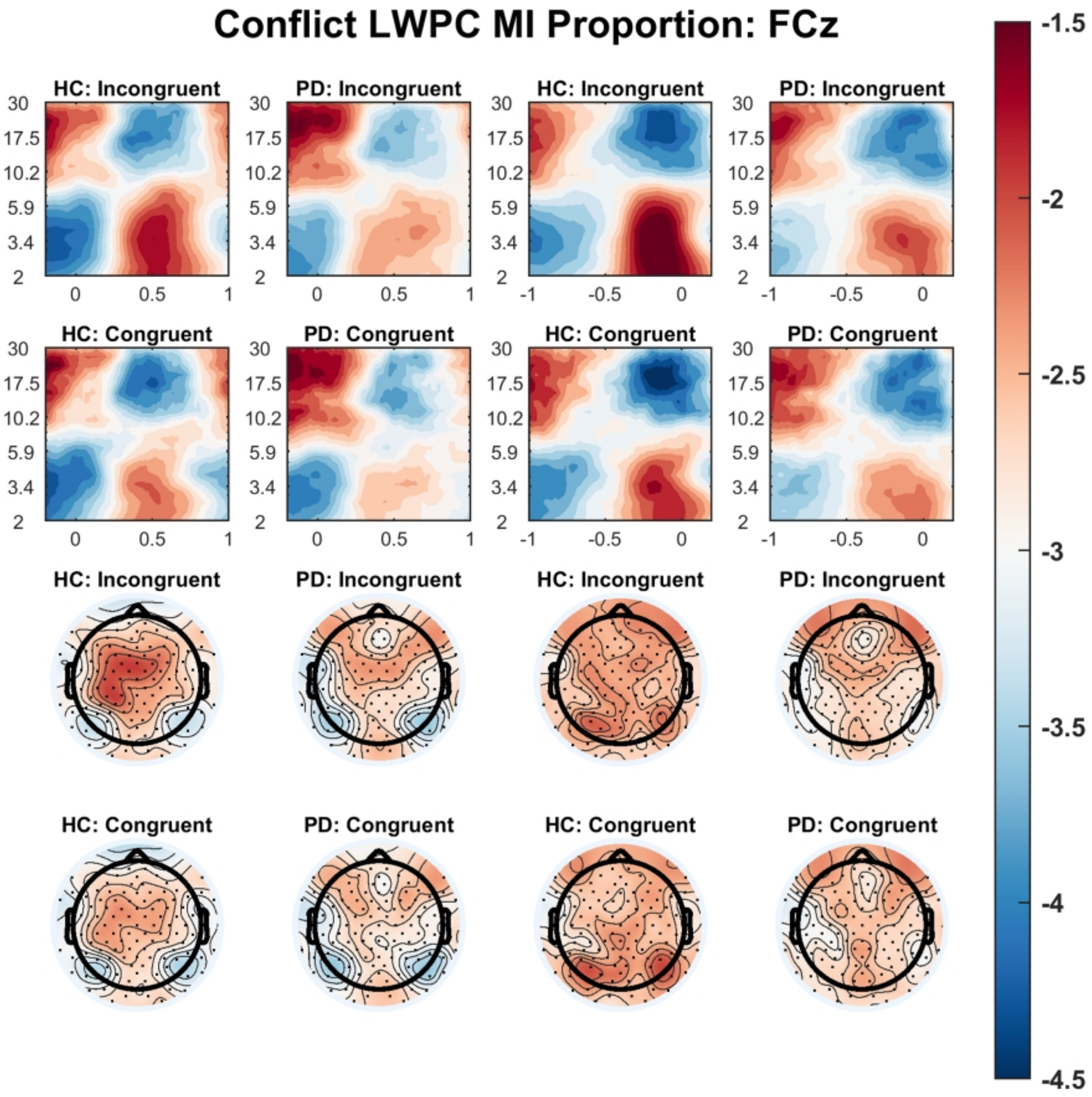

Supplementary Figure 4 Estimated marginal mean effects of the time-frequency regression analysis for the LWPCE manipulation of the mainly incongruent block. Data on the two left columns are plotted in reference to stimulus onset (SL), and data on the two right columns are referenced to the response (RL). The first two rows show the time-frequency results at channel FCz for incongruent and congruent items by group (HC and PD). The last two rows depict the averaged theta power between 0.3 and 0.7s SL and -0.6s to -0.2s RL by congruence and group.

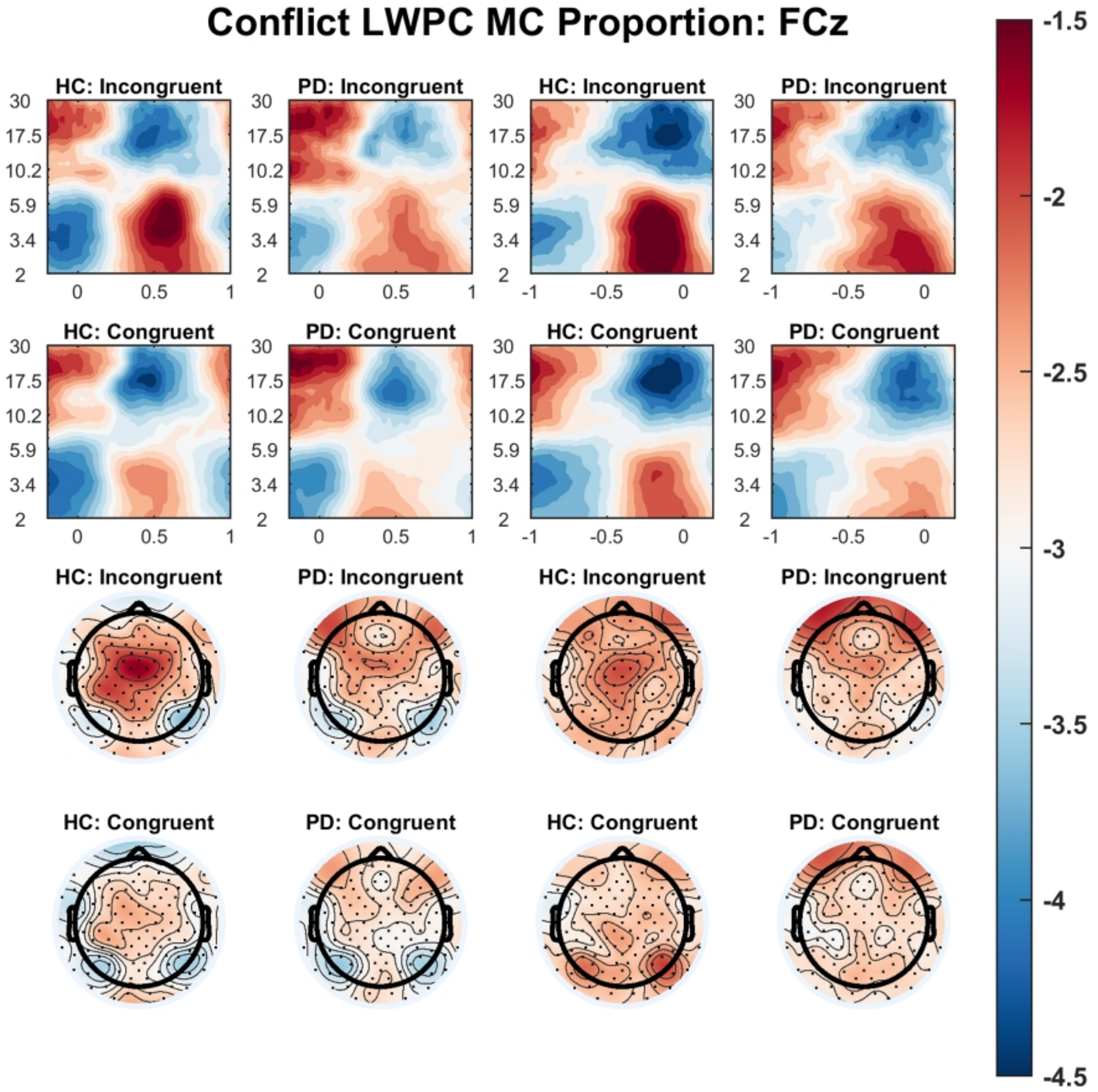

Supplementary Figure 5 Estimated marginal mean effects of the time-frequency regression analysis for the LWPCE manipulation of the mainly congruent block. Data on the two left columns are plotted in reference to stimulus onset (SL), and data on the two right columns are referenced to the response (RL). The first two rows show the time-frequency results at channel FCz for incongruent and congruent items by group (HC and PD). The last two rows depict the averaged theta power between 0.3 and 0.7s SL and -0.6s to -0.2s RL by congruence and group.

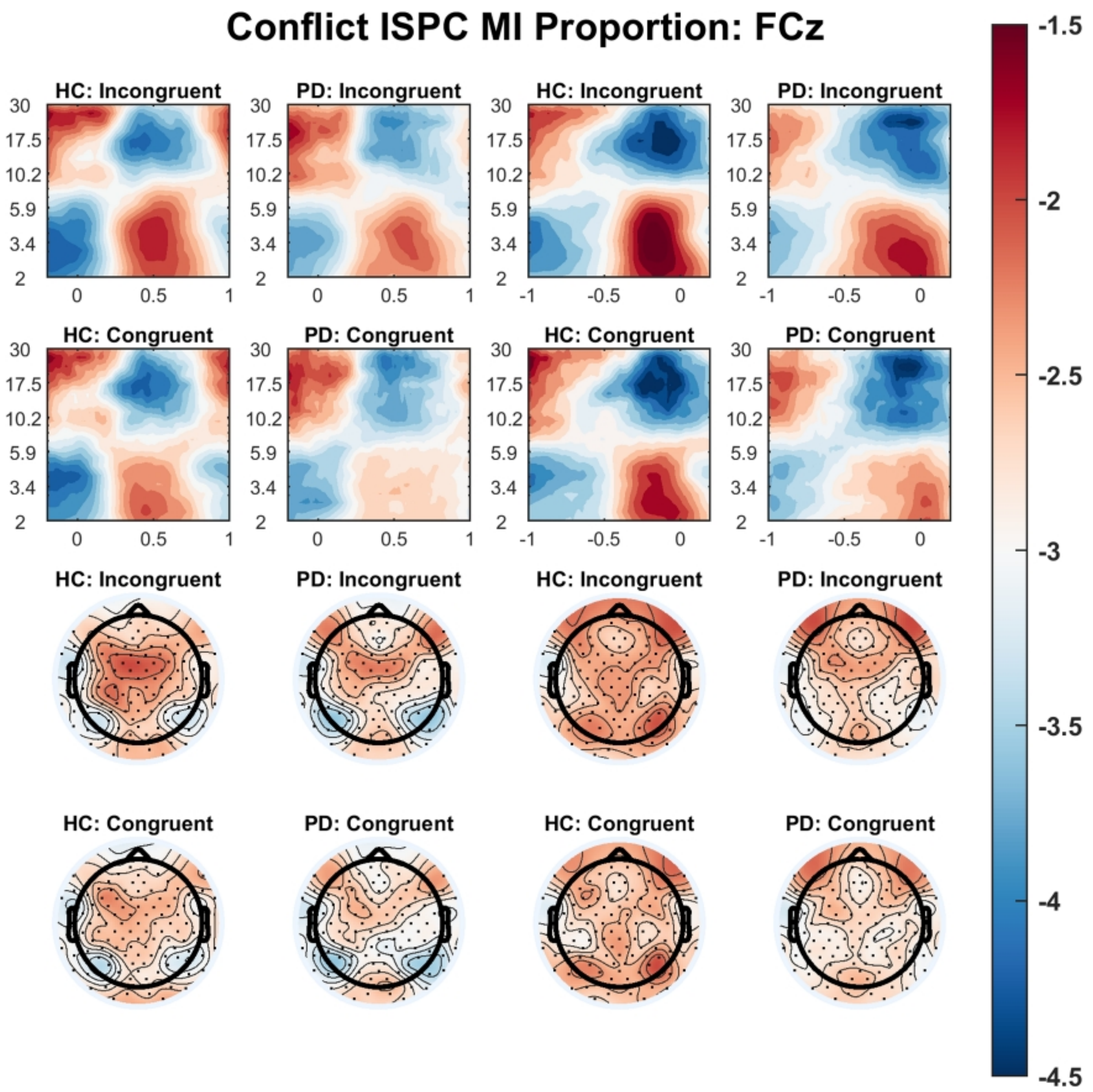

Supplementary Figure 6 Estimated marginal mean effects of the time-frequency regression analysis for the ISPCE manipulation of the mainly incongruent items. Data on the two left columns are plotted in reference to stimulus onset (SL), and data on the two right columns are referenced to the response (RL). The first two rows show the time-frequency results at channel FCz for incongruent and congruent items by group (HC and PD). The last two rows depict the averaged theta power between 0.3 and 0.7s SL and -0.6s to -0.2s RL by congruence and group.

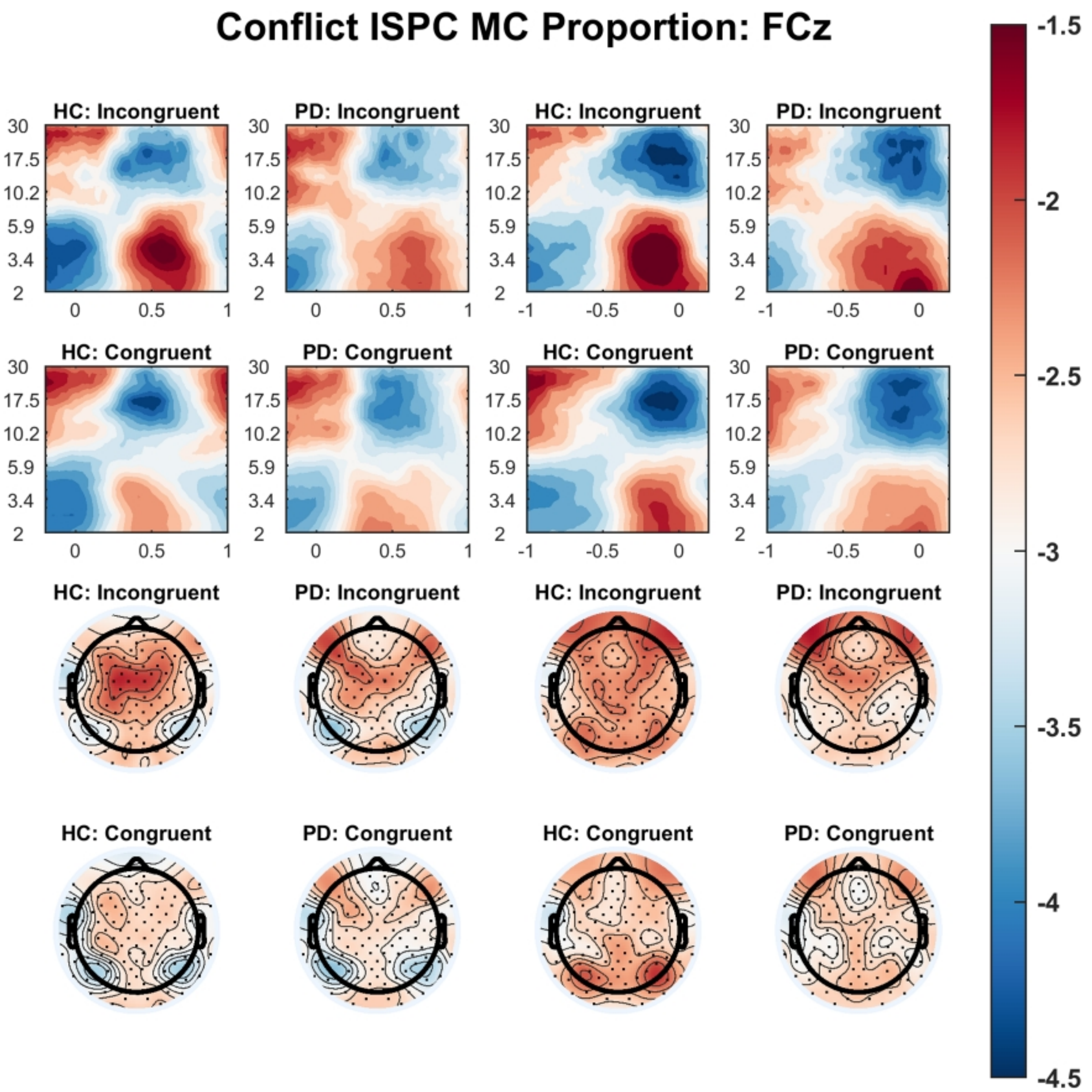

Supplementary Figure 7 Estimated marginal mean effects of the time-frequency regression analysis for the ISPCE manipulation of the mainly congruent items. Data on the two left columns are plotted in reference to stimulus onset (SL), and data on the two right columns are referenced to the response (RL). The first two rows show the time-frequency results at channel FCz for incongruent and congruent items by group (HC and PD). The last two rows depict the averaged theta power between 0.3 and 0.7s SL and -0.6s to -0.2s RL by congruence and group

References

1. Dadon G, Henik A. Adjustment of control in the numerical Stroop task. *Mem Cognit*. 2017;45(6):891-902. doi:10.3758/s13421-017-0703-6
